# Bovine oviductal organoids: a multi-omics approach to capture the cellular and extracellular molecular response of the oviduct to heat stress

**DOI:** 10.1186/s12864-023-09746-y

**Published:** 2023-10-27

**Authors:** Nico G. Menjivar, Ahmed Gad, Riley E. Thompson, Mindy A. Meyers, Fiona K. Hollinshead, Dawit Tesfaye

**Affiliations:** 1 Animal Reproduction and Biotechnology Laboratory (ARBL), Department of Biomedical Sciences, College of Veterinary Medicine and Biomedical Sciences, Colorado State University, 3107 Rampart Rd, Fort Collins, CO 80521 USA; 2 Department of Animal Production, Faculty of Agriculture, Cairo University, Giza, 12613 Egypt; 3 Animal Reproduction and Biotechnology Laboratory (ARBL), Department of Clinical Sciences, College of Veterinary Medicine and Biomedical Sciences, Colorado State University, Fort Collins, CO 80523 USA

**Keywords:** Organoids, Extracellular vesicles, Oviduct, Heat stress, Transcriptome, miRNA

## Abstract

**Background:**

The mammalian oviduct is a complex, fibromuscular organ known for its role in orchestrating a series of timely and dynamic changes to suitably support early embryogenesis. Climate change-induced heat stress (HS) is one of the largest single stressors compromising reproductive function in humans and farm animals via systemic changes in the redox status of the maternal environment, adversely affecting fertilization and early embryonic development. Oviductal organoids represent a unique 3-dimensional, biomimetic model to study the physiology of the oviduct and its subsequent impact on embryo development under various environmental conditions.

**Results:**

Our study is the first to demonstrate an innovative approach to understanding the cascade of molecular changes sustained by bovine oviductal organoids under HS and the subsequent maternal signals harnessed within their secreted extracellular vesicles (EVs). Transcriptomic analysis of oviductal organoids exposed to HS revealed 2,570 differentially expressed genes (1,222 up‐ and 1,348 downregulated), while EV-coupled miRNome analysis disclosed 18 miRNAs with significant differential expression (12 up- and 6 downregulated) in EVs from thermally stressed organoids compared to EVs released from organoids cultured under thermoneutral conditions. Genes activated in oviductal organoids in response to thermal stress, include: *COX1*, *ACTB*, *CST6*, *TPT1*, and *HSPB1*, while miR-1246, miR-148a, miR21-5p, miR-451, and miR-92a represent the top highly abundant EV-coupled miRNAs released in response to HS. Pathway analysis of genes enriched in organoids exposed to thermal stress showed the enrichment of endocrine resistance, cellular senescence, and notch signaling pathways. Similarly, EV-coupled miRNAs released from thermally stressed organoids showed their potential regulation of genes involved in cellular senescence, p53 signaling, and TGF-beta signaling pathways.

**Conclusions:**

In conclusion, the cellular and extracellular response of bovine oviductal organoids to in vitro HS conditions reveal the prospective impact of environmental HS on the physiology of the oviduct and the probable subsequent impacts on oocyte fertilization and early embryo development. Future studies elucidating the potential impact of HS-associated EVs from oviductal organoids on oocyte fertilization and preimplantation embryo development, would justify the use of an organoid model to optimally understand the oviduct-embryo communication under suboptimal environments.

**Supplementary Information:**

The online version contains supplementary material available at 10.1186/s12864-023-09746-y.

## Background

The mammalian oviduct comprises an advantageous microenvironment with biochemical and biophysical support to sustain the formation of life as the ‘site’ of fertilization and preimplantation embryo development [1]. Effective measures of communication are cohesively required from the embryo itself and the maternal environment to establish a periconceptional milieu to support conception and early embryonic development [2]. Tangible evidence exists demonstrating an unequivocal two-way signaling interaction at the embryo–maternal interface [3, 4], suggesting reciprocal crosstalk leading to a successful pregnancy provides a significant basis for physiological implantation [3–5]. Classically, cell–cell communications’ paramount role in regulating physiological processes were commonly defined either via direct contact through membrane signaling (juxtracrine) or by secreting various molecules (growth factors, hormones, cytokines, metabolites, etc.) with ensuing signaling function (autocrine, paracrine, and endocrine). More recently, extracellular vesicles (EVs) which exist in almost all biological fluids, have been implicated as emerging bi-directional, putative messengers to nano-shuttle regulatory bioactive molecules (lipids, proteins, nucleic acids, etc.) with biologically plausible functions [6–9]. Irrespective of their origin, previous reports have shown that EVs released following external stress stimuli induce bystander tolerance in naïve cells treated with ‘stressed EVs,’ [10–15] indicating their potential to transfer ‘protective’ molecules (protectosomes).

The large-scale impacts of elevated ambient temperatures resulting from climate change on animal production and reproductive performance have been extensively studied [16, 17]. External heat acting on a homeothermic animal impede fertility, affecting the physiological and cellular function of several reproductive tissues [18, 19]. In vivo and in vitro studies have demonstrated the direct effects of heat stress (HS)-induced dysfunction through impairments to mammalian oocyte maturation, fertilization, and early embryo development [20–23]. However, the complexity to investigate the impact of HS in regard to oviduct physiology and the embryo-oviduct dialog in later stage embryos have been largely stunted due to its inaccessible location without surgery or necropsy post-slaughter. Therefore, novel approaches to recapitulate the in vivo physiology and function of the oviduct in vitro, would provide an innovative mechanism to validate the system's functional response to HS.

Despite the extensive work carried out to understand the effects of HS on reproductive function, its underlying impacts on oviduct physiology remain largely unknown. Initial attempts to study the embryo-oviduct dialogue via EVs from oviductal fluid [24] and monolayer-bovine oviductal epithelial cell (BOEC) conditioned culture medium [25], demonstrated promising improvements in embryo quality and cryotolerance following their addition during embryo culture. Regardless of the beneficial roles of EVs, the potential transfer of identified embryotropic molecules eliciting such positive responses in embryo development was not studied. However, Almiñana and colleagues have shown major discrepancies in the protein cargo signatures from in vivo vs. in vitro derived EVs of oviductal origin, revealing that in vitro monolayer-BOECs inadequately mimic the in vivo oviductal environment, likely resulting from a lack of paracrine signaling amid reduced cellular diversity [26].

Organoids represent biomimetic, three-dimensional, in vitro cell clusters that organizationally self-regenerate into complex structures to mirror the physiological function of their tissue of origin [27]. Generally, organoid cell clusters are encapsulated within a jelly-like extracellular matrix and function similar to their native in vivo organ in terms of gene and protein expression, metabolic function and microscale tissue architecture [28]. Studies in humans [29] and mice [30, 31] have recently outlined advantages over existing traditional cell culture models through the establishment and long-term stability of fallopian tube organoids that faithfully recapitulate the phenotype of their in vivo tissue. Thus, in this study, we aimed to utilize bovine oviductal organoids to decrypt the oviducts’ differential response to HS in terms of cellular transcriptome and secreted EV-miRNome, to elucidate the potential downstream impacts on fertilization and early embryo development. Furthermore, several studies have revealed the regulatory role of miRNA sequence motifs and their corresponding RNA binding proteins in EV-miRNA secretion versus cellular retention under specific environmental conditions [32–36]. Therefore, investigating the interplay between miRNA sequence motifs and RNA binding proteins in the packaging and release of specific miRNAs into EVs by subjecting organoids to thermal stress, would shine light upon the molecular mechanisms behind the oviductal response to HS via EV-coupled miRNA signaling.

## Results

**Fig. 1:**
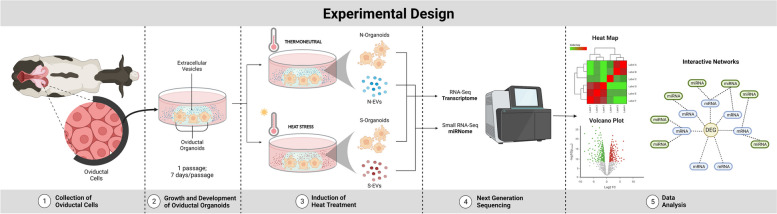
Schematic Illustration of the Experimental Design. Oviductal cells were collected from abattoir-derived reproductive material for the establishment of bovine oviductal organoids. Organoids were subsequently grown and developed under thermoneutral conditions for 14 days prior to exposing to heat treatment. Following HS exposure, organoid cells and the corresponding EVs were collected, isolated and subjected to genome-wide transcriptomic and miRNomic profiling, respectively

The experimental design outlining the different groups of organoid cultures and EVs used in the RNA-seq analyses are summarized in Fig. 1.

### 3-D matrix supports dynamic oviductal organoid culture and EV production

**Fig. 2:**
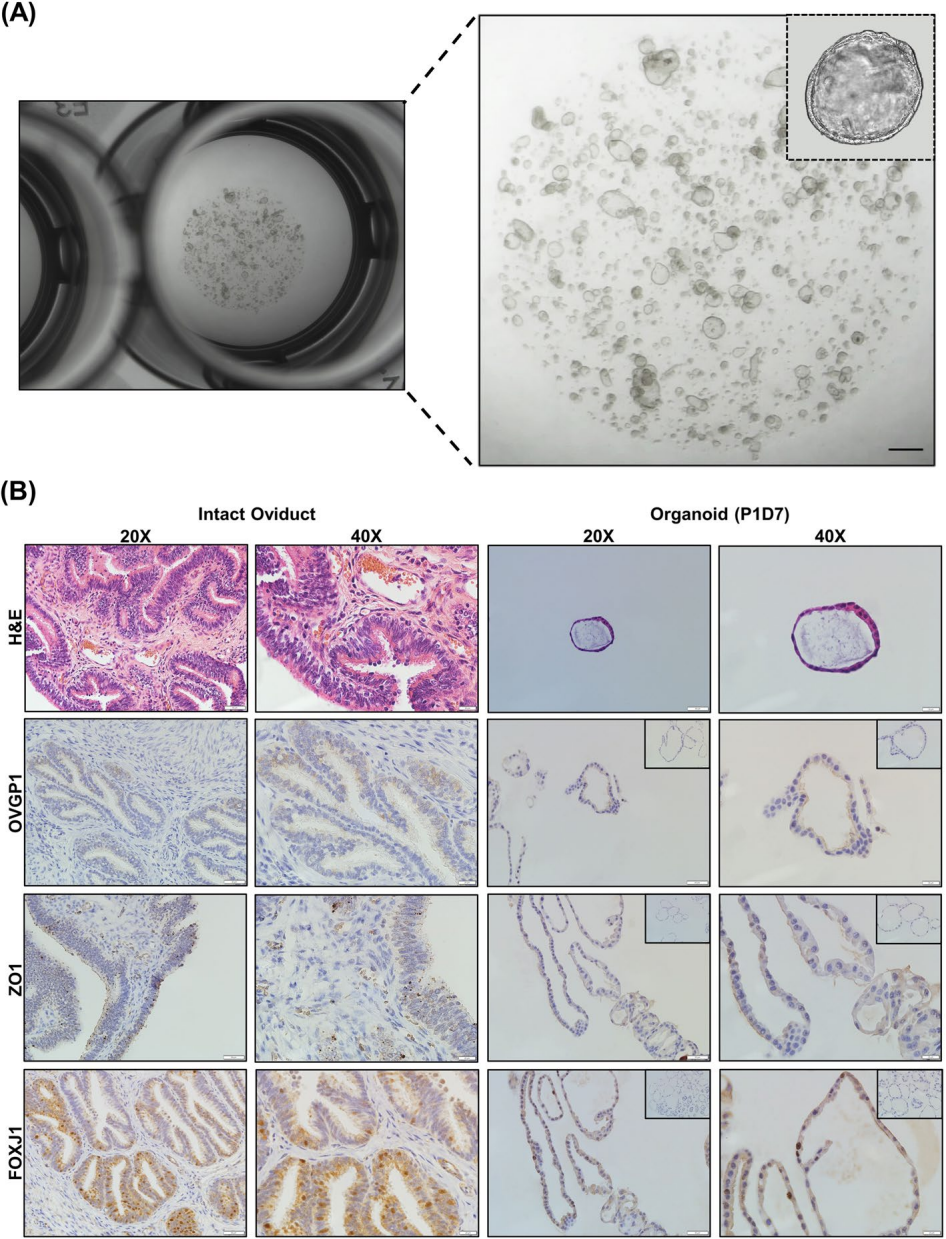
Establishment of Oviductal Organoid Culture System. Representative morphology of cultured bovine oviductal organoids after 14 days in culture. Scale bar, 1 mm (**A**). Immunostaining of in vitro cultured bovine oviductal organoids at passage 1, day 7 (P1D7). Histological oviductal samples with H&E staining, and immunohistochemical (OVGP1, ZO1, and FOXJ1) at 20 × and 40 × magnification, respectively. Scale bar = 50 μm at 20 × magnification and 20 μm at 40 × magnification. Controls shown in insets represent a negative reagent control (OVGP1, ZO1 and FOXJ1)

**Fig. 3:**
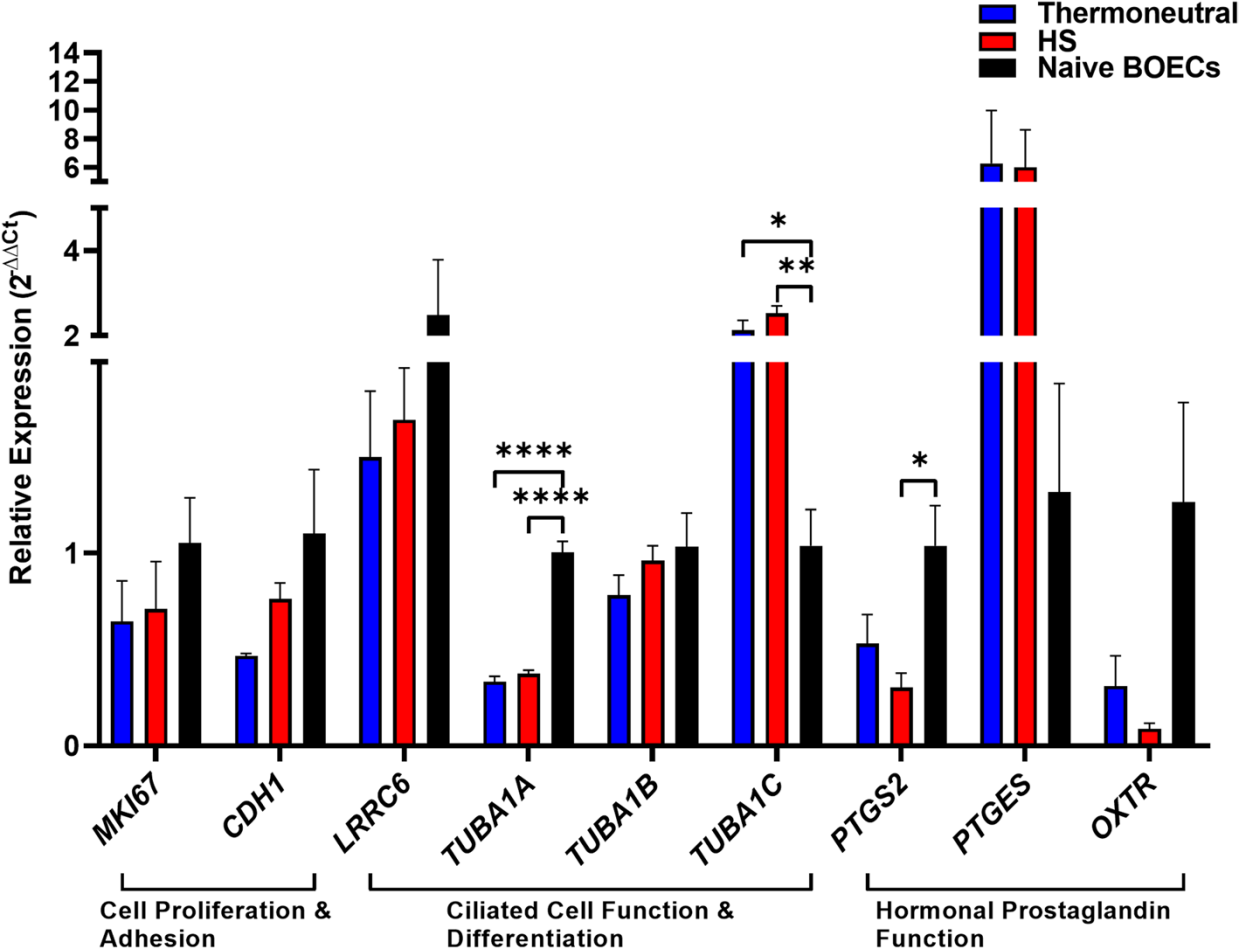
In vitro Produced Organoid Retention of the In vivo Oviduct. Quantitative real time PCR analysis of selected genes outlining the mRNA expression of markers for cell proliferation and adhesion, ciliated cell function and differentiation, and hormonal prostaglandin function in in vitro produced organoids (37 °C and 42 °C) and naïve bovine oviductal epithelial cells (BOEC). Data are shown as the mean ± SEM and the differences between means were analyzed using one-way ANOVA followed by Tukey’s Multiple Comparisons Test in biologically independent samples (*n* = 3). Statistical significance (*) between groups were determined at (*p* < 0.05)

**Fig. 4:**
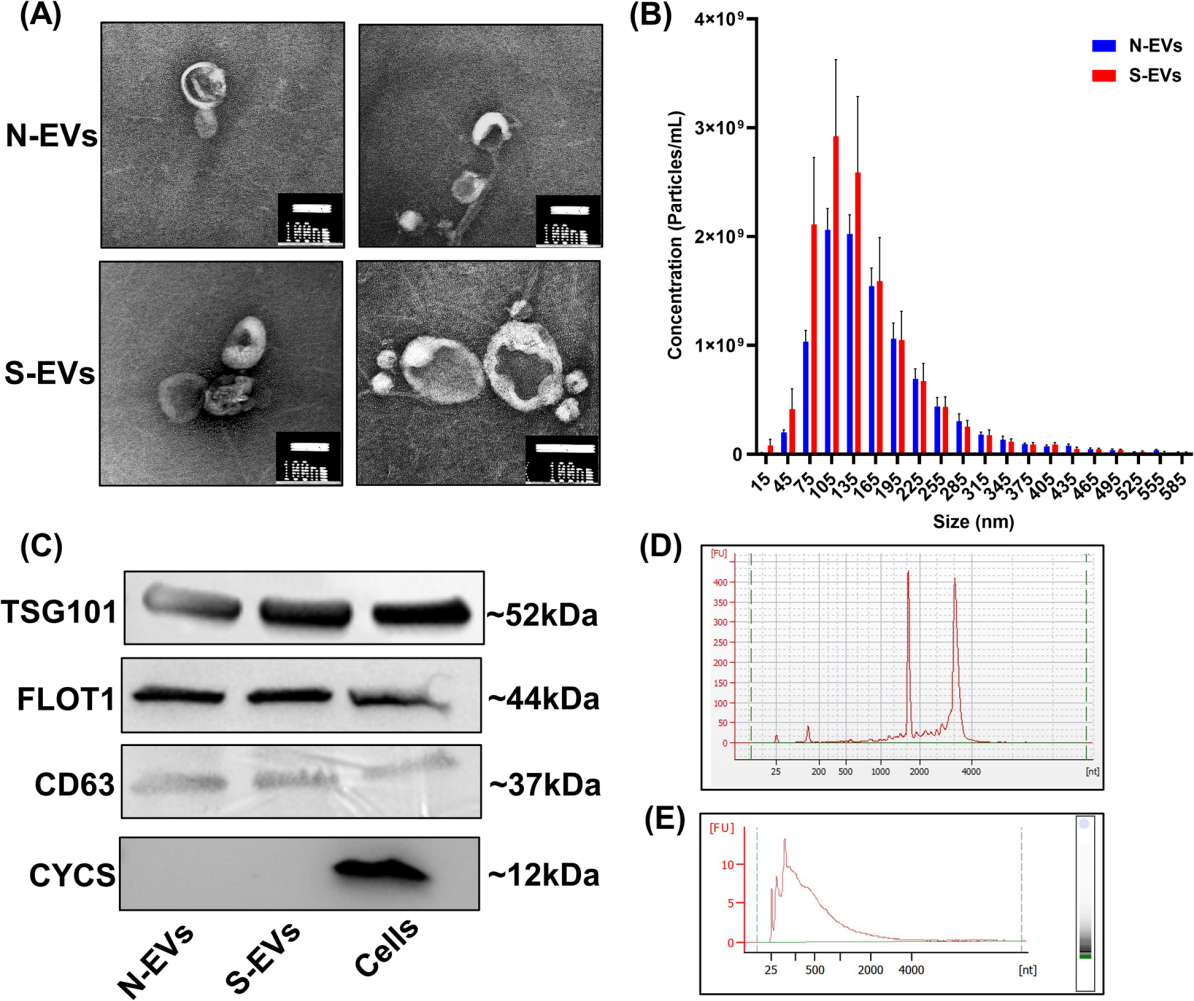
Characterization of Oviductal Organoid-derived EVs. Transmission Electron Microscopy (TEM) images depicting a clear bilipid enclosed complex in oviductal organoid-derived EVs. Scale bar, 100 nm (**A**). Size distribution and concentration of N-EVs (blue bars) and S-EVs (red bars) resulting from Nanoparticle Tracking Analysis (NTA) (**B**). Cropped blots for Western Blotting (WB) analysis in EVs shows traditionally expressed EV-associated protein markers (TSG101, FLOT1, CD63) and the absence of expression for non-EV, mitochondrial associated cellular marker protein (CYCS) in lanes indicated by N-EVs and S-EVs (**C**). Representative RNA quality control and size distribution from oviductal organoids (**D**) and the corresponding EVs indicating the absence of 18 s and 28 s cellular RNA components (**E**)

Oviductal organoids generated from bovine oviducts resected from diestrus reproductive tracts (*n* = 10) were grown for 14 days (one passage, 7 days per passage; Fig. 2A), prior to suspension in a xeno-free, synthetic matrix for experiments with HS. Representative histological and immunohistochemical (Fig. 2B) images of in tact oviduct and in vitro cultured oviductal organoids (P1D7) demonstrate the architectural morphology (hematoxylin and eosin; H&E) and presence of oviduct-specific glycoprotein 1 (OVPG1), zonula occludens-1 (ZO1), and forkhead box J1 (FOXJ1), respectively. H&E staining showed that crosssections of oviductal organoids contain a monolayer of oviductal epithelial cells encircling a centralized lumen. Also, mature organoids maintained through extended culture persistently express hallmark protein OVGP1, as well as epithelial cell tight junctions (ZO1), and ciliated cells (FOXJ1). Comprehensively, the organoid cellular morphology contain both partially differentiated and polarized cuboidal cells, as well as columnar shaped cells, which precisely represent the established structure of functional organoids. Additionally, we used a gene panel to functionally characterize the expression patterns of in vitro produced oviductal organoids (37 °C and 42 °C) compared to naïve in vivo BOECs. The panel was comprised of cell proliferation and adhesion (*MKI67* and *CDH1*), ciliated cell function and differentiation (*LRRC6*, *TUBA1A*, *TUBA1B* and *TUBA1C*), and hormonal prostaglandin function (*PTGS2*, *PTGES* and *OXTR*) gene expression signatures. Overall, minimal statistical differences presided between in vitro produced organoids and in vivo derived cells, strengthening the biomimicry of extended culture in a 3-D matrix (Fig. 3). In addition, a dual-targeted approach to isolate EVs using ultracentrifugation and size exclusion chromatography (SEC) was employed to isolate a relatively homogenous population of oviductal organoid-derived EVs. The molecular and morphological characterization of oviductal organoid-derived EVs validated the integrity of our procedure to isolate molecular analysis grade EVs from conditioned culture medium of in vitro-cultured bovine oviductal organoids. Representative TEM images (Fig. 4A), indicate the presence of membrane-enveloped vesicles released from oviductal organoids. Moreover, nanoparticle tracking analysis revealed a higher particle concentration in the S-EVs (derived from organoids cultured at 42 °C; 1.29E + 10 particles/mL), when compared to the N-EVs (derived from organoids cultured at 37 °C; 1.01E + 10 particles/mL), with median sizes of 120.01 and 133.99 nm, respectively (Fig. 4B). Western blot analysis (Fig. 4C) indicated the presence of EV marker proteins (TSG101, FLOT1, and CD63) in the EV samples derived from oviductal organoids, while CYCS, a cellular contaminant and negative marker of EVs, was absent. In addition, RNA quality control for integrity, prior to RNA sequencing, revealed the presence of 18S and 28S ribosomal RNA peaks in the oviductal organoid RNA and their absence in oviductal organoid-derived EV-RNA samples (Fig. 4D & E).

### Decoding the transcriptome response of oviductal organoids to HS

**Fig. 5:**
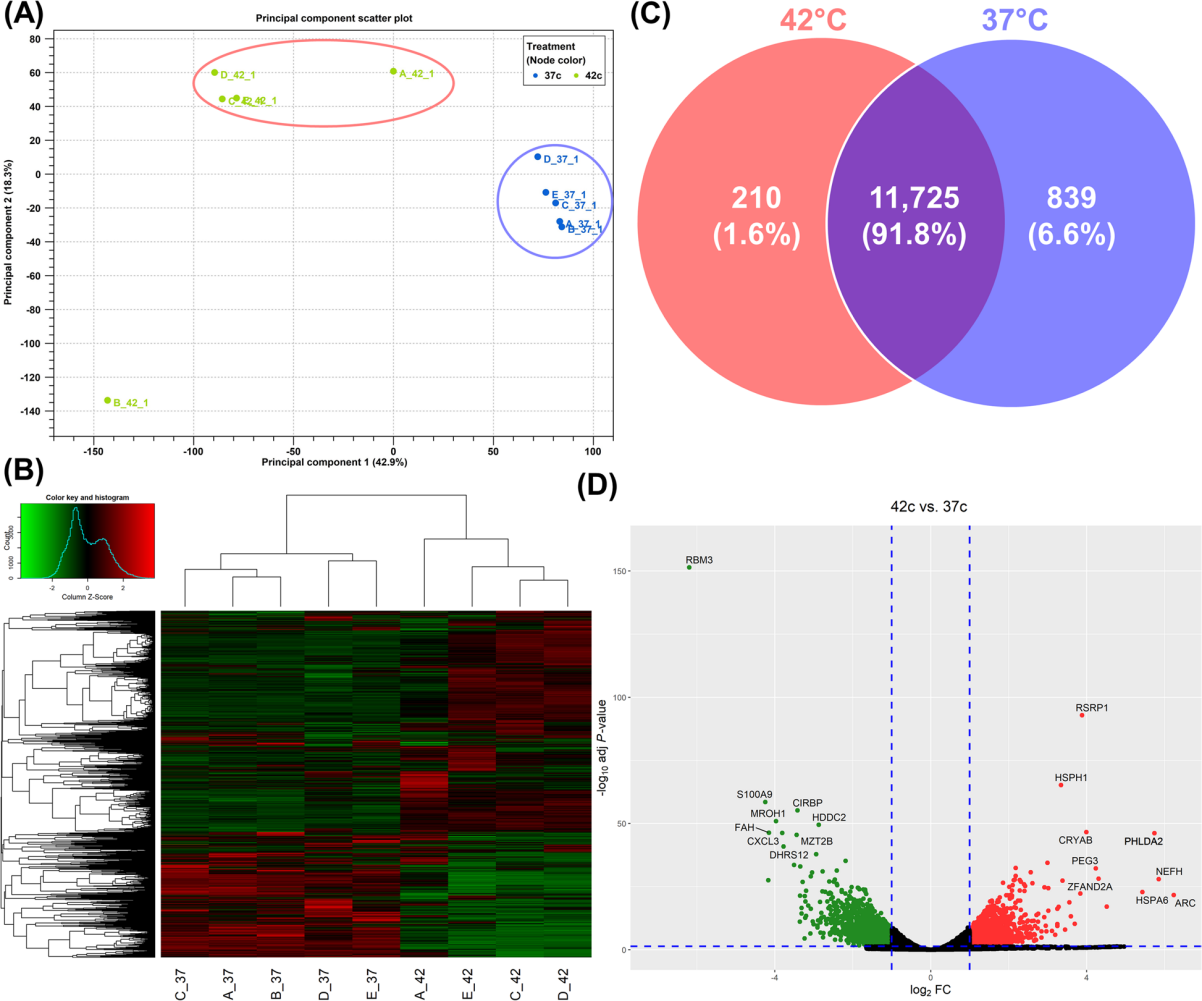
Impact of HS on Oviductal Organoids: Transcriptomic Cellular Response. Principal component analysis (PCA) (**A**) and heatmap/hierarchical clustering of oviductal organoid samples (**B**) cultured under HS (42 °C; A_42-D_42) or thermoneutral conditions (37 °C; A_37-D_37). Venn diagram indicating exclusively and commonly expressed genes in the 42 °C and 37 °C groups (**C**). Volcano plot displaying differentially expressed genes, up- and downregulated in oviductal organoids cultured under HS (42 °C) compared to those cultured under thermoneutral conditions (37 °C) (**D**)

To understand the changes in molecular physiology of the bovine oviduct under HS conditions, this study examines the first comprehensive transcriptome analysis revealing the cellular response of oviductal organoids to elevated thermal stress. A total of ten RNA libraries were prepared from the organoid cells cultured under HS (42 °C) and thermoneutral conditions (37 °C). Approximately 536 million raw reads were sequenced from both groups. After adapter trimming and quality filtering, approximately 53.6 million reads per library were retained with an average of 99% of the quality-controlled (QC) reads mapped to the bovine reference genome (ARS-UCD1.2). A summary of the total number of reads and the mapping results for each group is presented in Additional file 1: Table S1. Principal component analysis (PCA) and the accompanied hierarchical heatmap revealed that biological replicates for each group clustered together except for one replicate amid the HS groups (Fig. 5A & B), which has been excluded from further analysis. A stringent criterion was set to avoid bias for low expression in which, expressed genes represent zFPKM >  − 3 amongst all replicates. In total, 11,935 and 12,564 genes were detected in the HS and thermoneutral cultured organoid groups, respectively, with 11,725 genes mutually expressed in both groups (Fig. 5C). Of the most highly expressed genes in both groups, the top 20 genes most abundantly expressed amongst all organoid samples include: *COX1*, *ACTB*, *CST6*, *TPT1*, *RPLP1*, *RPLP0*, *FTH1*, *COX2*, *EEF1A1*, *UBA52*, *RPS15* (Table 1).

**Table 1:** Top 20 expressed genes in oviductal organoids from the HS (42 °C) and thermoneutral (37 °C) groups

Genes	42 °C Group TPM	%^a^	Genes	37 °C Group TPM	%^a^
*COX1*	12,134.48	10.36	*COX1*	18,703.53	14.63
*ACTB*	9851.64	8.41	*COX3*	12,044.18	9.42
*CST6*	7866.93	6.71	*COX2*	9073.11	7.10
*TPT1*	6996.46	5.97	*FTH1*	8633.91	6.76
*HSPB1*	6814.69	5.82	*RPLP1*	6970.11	5.45
*RPLP1*	6408.56	5.47	*GAPDH*	6133.64	4.80
*RPLP0*	6155.16	5.25	*RPLP0*	5702.06	4.46
*CLU*	5635.77	4.81	*ATP6*	5258.80	4.11
*CRYAB*	5519.48	4.71	*KRT19*	5247.57	4.11
*FTL*	5403.90	4.61	*FTL*	5217.36	4.08
*ACTG1*	5399.25	4.61	*TPT1*	5153.81	4.03
*COX3*	5017.02	4.28	*ACTB*	4863.59	3.81
*FTH1*	5004.65	4.27	*ACTG1*	4709.11	3.68
*COX2*	4958.39	4.23	*EEF1A1*	4561.17	3.57
*EEF1A1*	4524.72	3.86	*RPS15*	4490.25	3.51
*UBA52*	4411.87	3.77	*CST6*	4456.38	3.49
*HSPA1A*	4367.03	3.73	*ND3*	4434.30	3.47
*ANXA2*	3736.05	3.19	*ND1*	4104.32	3.21
*HSPA8*	3531.16	3.01	*MSLN*	4025.36	3.15
*RPS15*	3418.48	2.92	*UBA52*	4018.46	3.14

*TPM* average Transcript Per Million mapped reads

^a^Percentage of the genes sequence reads

**Fig. 6:**
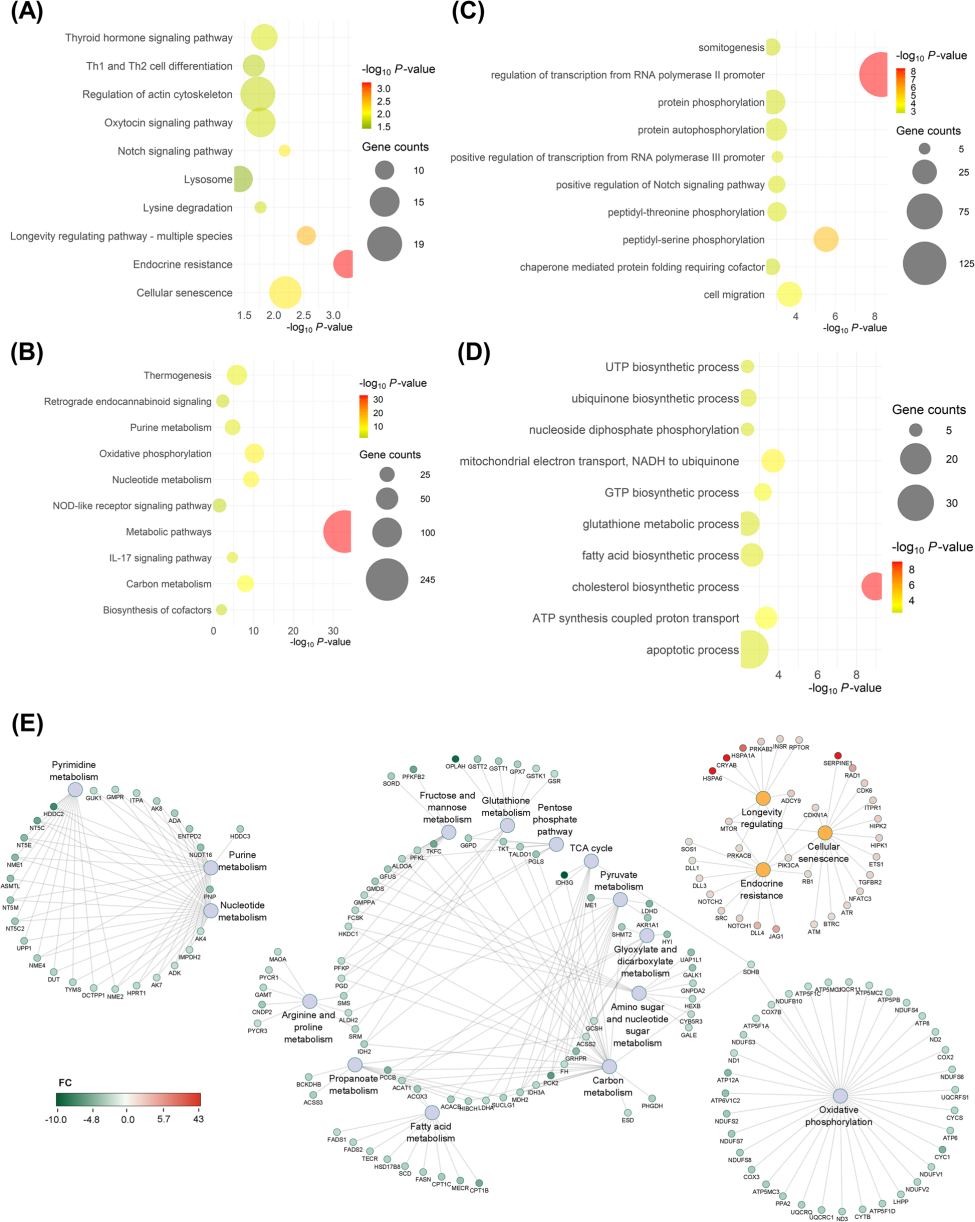
Oviductal Organoids: Ontological Classification and Interactive Networking. Bubble plots, revealing the top 10 pathways and biological processes targeted by the up- (**A** and **C**) and downregulated genes (**B** and **D**) in the 42 °C compared to the 37 °C groups, respectively. The color and size of each bubble represent the P-value and number of gene counts. Interactive networking of the differentially expressed genes commonly involved in highly enriched pathways including the up- (red) and downregulated (green) genes in the 42 °C compared to the 37 °C groups (**E**)

Considering a false discovery rate (FDR) < 0.05 and a fold change (FC) ≥ 2, a total of 2,570 differentially expressed genes (DEGs) (1,222 up‐ and 1,348 downregulated) were identified in the oviductal organoids subjected to HS compared to the thermoneutral groups, including 170 genes with unknown function (Additional file 2: Table S2). The volcano plot (Fig. 5D) illustrates the distribution of DEGs, up- and downregulated, in the HS compared to the thermoneutral groups. The top 20 known DEGs (upregulated include: *CRISPLD2*, *HSPA6*, *SERPINE1*; downregulated include: *RBM3*, *MROH1*, *FAH*) identified in the HS compared with the thermoneutral cultured groups are listed in Table 2. Gene ontology (GO) enrichment analysis of the upregulated genes in the HS groups revealed that endocrine resistance, cellular senescence, and notch signaling pathway to be the top significant pathways (Fig. 6A; Additional file 3A: Table S3A). Alternatively, metabolic pathways, oxidative phosphorylation, nucleotide metabolism, carbon metabolism, and thermogenesis were the top significant pathways dominated by the downregulated genes (Fig. 6B; Additional file 3B: Table S3B). Biological processes in which genes upregulated in thermally stressed organoids involve cell migration, transcription regulation, and protein phosphorylation (Fig. 6C; Additional file 4A: Table S4A), while cholesterol biosynthetic processes, mitochondrial electron transport and ATP synthesis were the processes in which involve downregulated genes (Fig. 6D; Additional file 4B: Table S4B). Interaction networks of DEGs commonly involved in highly enriched pathways are presented in Fig. 6E. The highlighted genes amid the interaction networks are involved in various signaling pathways, gene expression regulation, and functions regulating cellular senescence and pregnancy homeostasis.

**Table 2:** Top 20 DEGs in HS (42 °C) oviductal organoids compared to the thermoneutral (37 °C) groups

Upregulated Genes	FC	FDR	Downregulated Genes	FC	FDR
*RAB39A*	85.71	3.56E-03	*MFAP4*	-14.38	3.37E-11
*ARC*	75.04	1.91E-22	*PPP1R32*	-15.65	1.65E-09
*CRISPLD2*	64.85	1.25E-32	*MROH1*	-15.68	1.10E-51
*NEFH*	57.56	9.69E-29	*ACSM1*	-17.54	8.85E-19
*PHLDA2*	53.28	6.75E-47	*FAH*	-17.80	4.22E-47
*ENSBTAG00000001219*	52.94	1.64E-08	*RHBDD3*	-18.01	2.75E-28
*HSPA6*	42.95	1.46E-23	*NOS2*	-18.02	1.52E-19
*ENSBTAG00000052847*	36.38	1.28E-07	*S100A9*	-18.97	2.65E-59
*PURG*	31.95	3.79E-09	*ASB9*	-19.67	1.96E-11
*FAM71F1*	26.14	2.53E-08	*HERC3*	-19.85	6.46E-60
*NPB*	22.86	8.62E-18	*DIRAS3*	-21.65	3.80E-10
*GAS2*	20.03	9.82E-10	*HNMT*	-22.45	2.36E-10
*ZFAND2A*	19.72	6.73E-29	*ETNPPL*	-24.09	5.00E-10
*PEG3*	18.76	5.88E-33	*SERPINB13*	-32.57	9.11E-15
*CRYAB*	15.90	2.22E-47	*ENSBTAG00000003661*	-32.99	1.55E-20
*ENSBTAG00000049003*	14.77	2.45E-16	*CSF3*	-34.72	2.48E-17
*RSRP1*	14.73	1.16E-93	*LRCOL1*	-36.95	1.50E-05
*SERPINE1*	14.29	5.61E-23	*DAPL1*	-60.38	1.61E-08
*ENSBTAG00000050861*	12.67	2.26E-06	*RBM3*	-73.40	3.10E-152
*TEX49*	12.64	9.61E-08	*SERPINE3*	-163.84	1.87E-63

*FC* Fold Change, *FDR* False Discovery Rate

### HS alters oviductal organoid-derived EV-coupled miRNAs

**Fig. 7:**
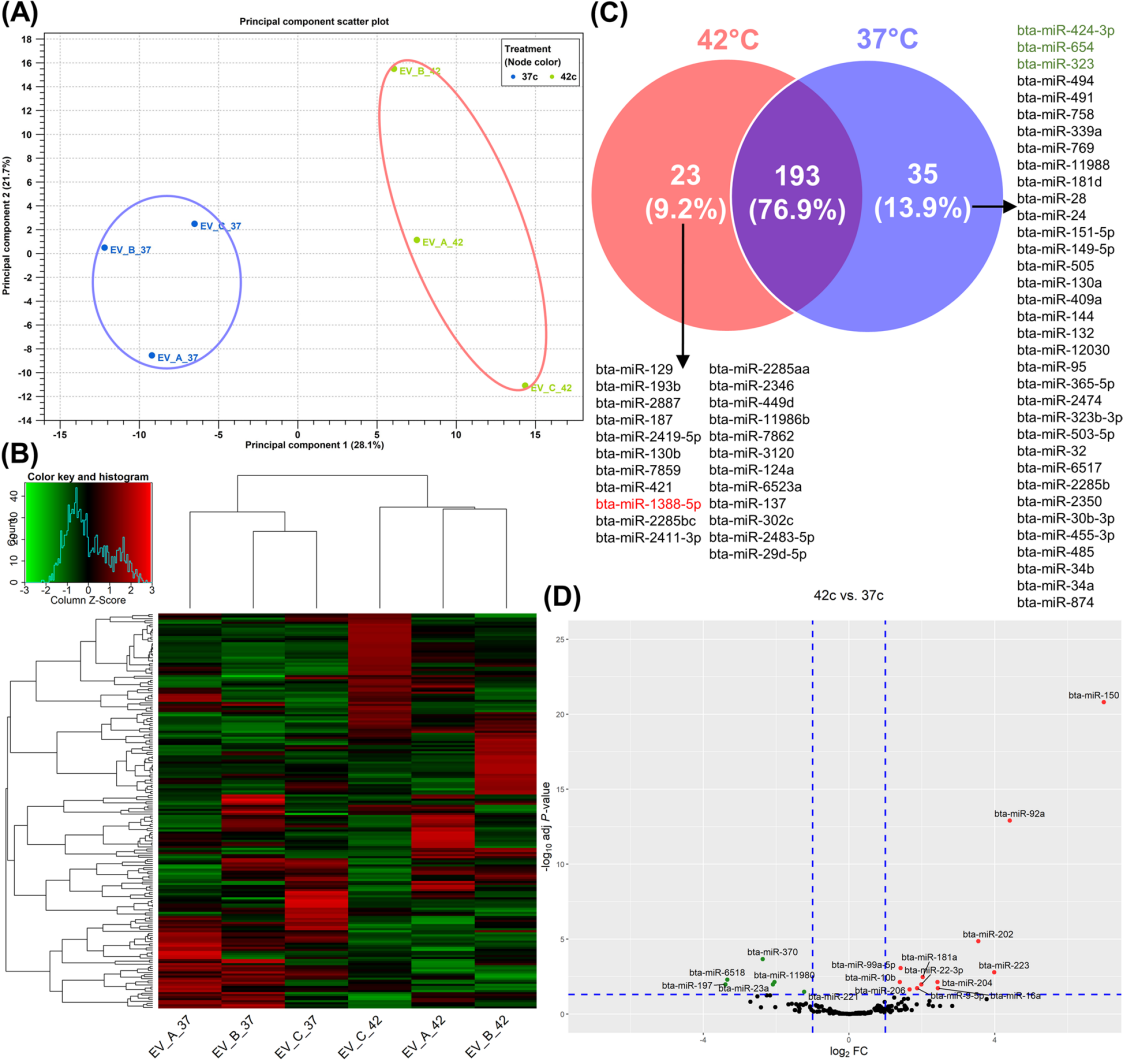
HS Alters Oviductal Organoid-derived EVs Molecular Cargo: miRNome Extracellular Response. Principal component analysis (PCA) (**A**) and heatmap/hierarchical clustering of oviductal organoid-derived EVs (**B**). Venn diagram indicating exclusively and commonly expressed miRNAs in the 42 °C and 37 °C groups (**C**), red and green miRNAs are significantly different (FDR < 0.05) between the two groups. Volcano plot displaying differentially expressed miRNAs, up- (red) and downregulated (green) in oviductal organoid-derived EVs from the 42 °C compared to 37 °C groups (**D**)

To understand the extracellular response of oviductal organoids to HS, organoid-secreted EVs were investigated for their miRNA content using small RNA sequencing technology. Three small RNA libraries were prepared from the EVs derived from each of the organoid cultures under 37 °C and 42 °C. Approximately 135 million raw reads were sequenced from both EV groups. After adapter trimming and quality control, an average of 21 million reads per library was retained. An average of 87% and 89% of these reads from the HS and thermoneutral EV groups, respectively, were mapped to the bovine reference genome (Additional file 5: Table S5). Based on the miRNA expression patterns, the PCA (Fig. 7A) and hierarchical heatmap (Fig. 7B) exhibited a clear clustering of the replicates from each group. Sequencing data analysis revealed a total of 251 known miRNAs amongst both groups with at least 10 counts per million (CPM) (Fig. 7C). While a complete list of all the expressed miRNAs detected is presented in Additional file 6: Table S6, a list of the top 20 expressed miRNAs from each group are presented in Table 3. Among the top 20 expressed miRNAs, 15 were commonly detected amongst both groups.

**Table 3:** Top 20 expressed miRNAs in oviductal organoid-derived EVs from the HS (42 °C) and thermoneutral (37 °C) groups

miRNA	42 °C Group CPM	%^a^	miRNA	37 °C Group CPM	%^a^
bta-miR-1246	714,796.52	52.39	bta-miR-1246	363,004.63	44.35
bta-miR-148a	91,700.77	6.72	bta-miR-148a	63,584.52	7.77
bta-miR-21-5p	66,565.11	4.88	bta-miR-21-5p	43,902.13	5.36
bta-miR-451	63,787.82	4.68	bta-let-7a-5p	38,199.05	4.67
bta-miR-92a	52,778.22	3.87	bta-miR-378	33,327.83	4.07
bta-miR-122	39,526.19	2.90	bta-miR-451	26,116.20	3.19
bta-let-7a-5p	35,926.19	2.63	bta-let-7i	22,986.77	2.81
bta-let-7i	25,351.78	1.86	bta-let-7f	20,881.75	2.55
bta-miR-378	23,826.87	1.75	bta-miR-122	19,106.09	2.33
bta-let-7f	19,848.71	1.45	bta-let-7b	17,898.58	2.19
bta-miR-26a	19,314.54	1.42	bta-miR-26a	16,336.63	2.00
bta-let-7b	17,836.14	1.31	bta-miR-151-3p	10,162.43	1.24
bta-miR-10b	12,289.04	0.90	bta-miR-200b	8044.04	0.98
bta-miR-151-3p	12,105.22	0.89	bta-miR-320a	6426.09	0.79
bta-miR-99a-5p	10,290.88	0.75	bta-miR-200c	5618.62	0.69
bta-miR-200b	9131.74	0.67	bta-let-7 g	5587.82	0.68
bta-miR-10a	7387.11	0.54	bta-miR-100	5124.45	0.63
bta-miR-143	6627.53	0.49	bta-miR-30d	5002.99	0.61
bta-miR-30d	6243.01	0.46	bta-miR-10b	4666.79	0.57
bta-miR-486	6147.07	0.45	bta-miR-192	4474.81	0.55

*CPM* average Counts Per Million mapped reads

^a^Percentage of the miRNA sequence reads

**Fig. 8:**
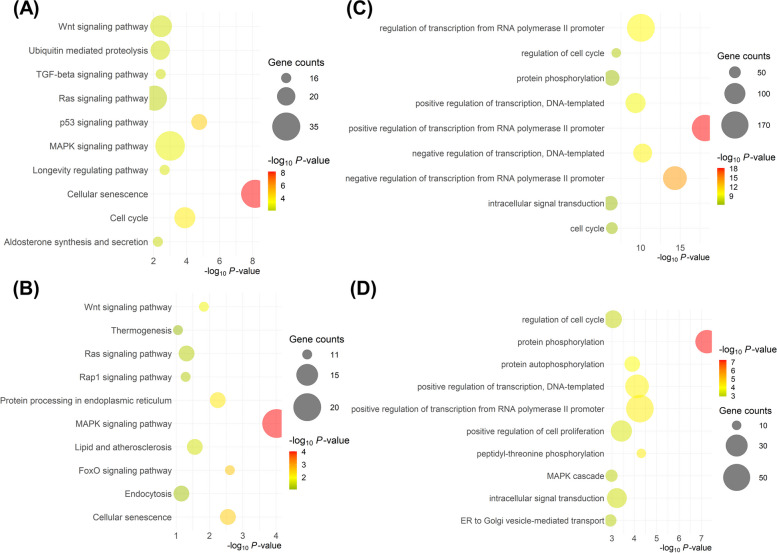
Oviductal Organoid-derived EV-miRNAs: Ontological Classification. Bubble plots, revealing the top 10 pathways and biological processes targeted by the up- (**A** and **C**) and downregulated miRNAs (**B** and **D**), respectively in the 42 °C compared to the 37 °C groups. The color and size of each bubble represent the P-value and number of miRNA target genes, respectively

**Fig. 9:**
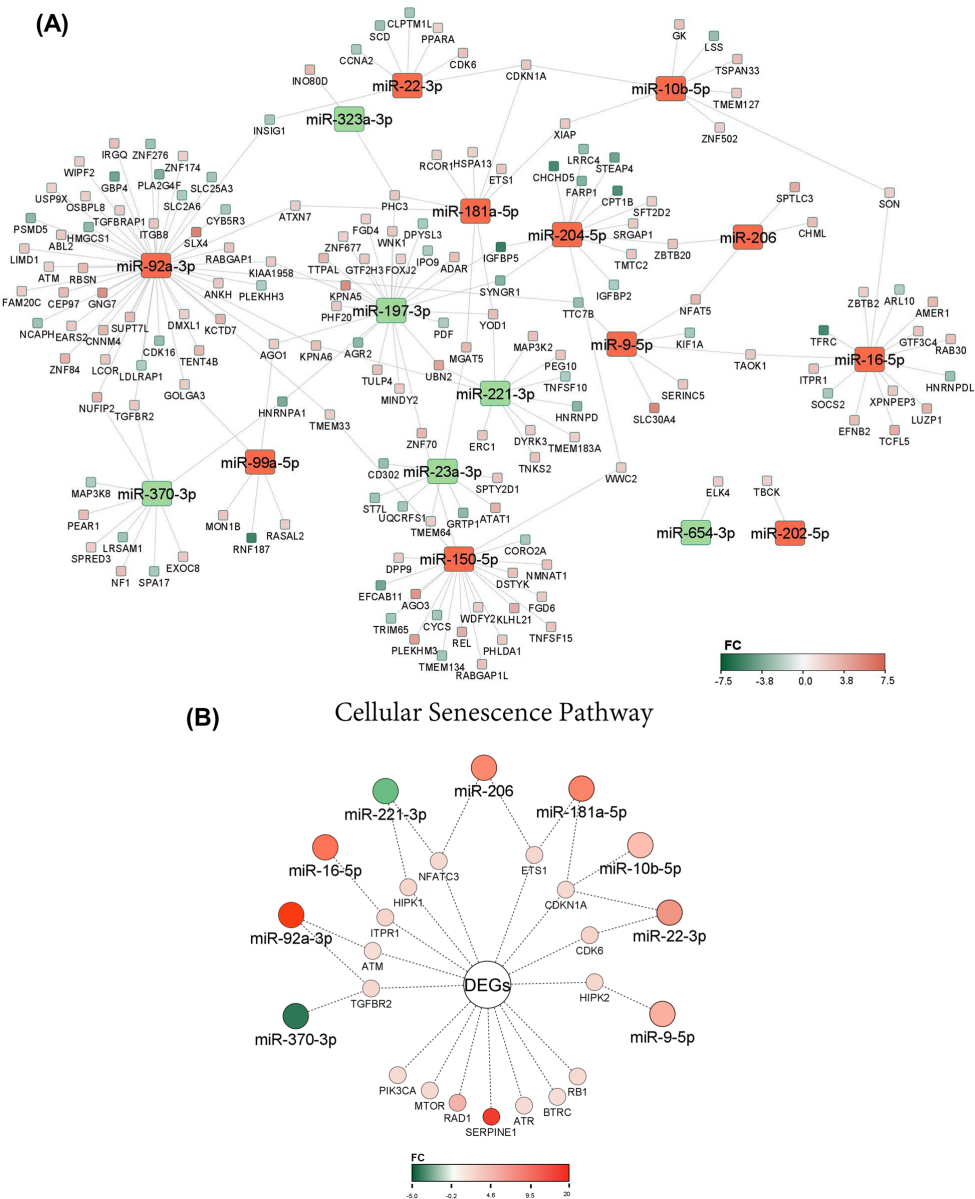
Oviductal Organoid DE-EV-miRNAs and DEGs Interaction Network. Interactive networking of common experimentally validated target genes of the DE-EV-miRNAs and the organoids DEGs in the 42 °C compared to the 37 °C groups (**A**). A network of the DEGs involved in the cellular senescence pathway and targeted by the DE-EV-miRNAs (**B**)

Differential expression analysis of EV-miRNAs released from oviductal organoids subjected to divergent ambient temperatures indicated a total of 18 miRNAs as significantly-differentially expressed between the two groups (FC > 2, FDR < 0.05). EVs from HS oviductal organoids exhibited the enrichment of 12 miRNAs (including: miR-150, miR-92a, miR-223, and miR-202) and 6 downregulated miRNAs (including: miR-221, miR-11980, miR-23a and miR-370) compared to the EVs released from thermoneutral cultured organoids (Table 4 and Fig. 7D). Interestingly, two differentially expressed (DE)-miRNAs (miR-10b and miR-92a) were also noted in the top 20 expressed miRNAs list. Pathway analysis revealed that genes potentially targeted by the upregulated EV-miRNAs were involved in cellular senescence, p53, and TGF-beta signaling pathways (Fig. 8A; Additional file 7A: Table S7A), while those potentially targeted by the downregulated EV-miRNAs were involved in MAPK and FoxO signaling pathways (Fig. 8B; Additional file 7B: Table S7B). Transcription and cell cycle regulation were among the top abundant biological processes targeted by the upregulated miRNAs (Fig. 8C; Additional file 8A: Table S8A), while protein phosphorylation and regulation of cell cycle and proliferation were among the top biological processes targeted by the downregulated miRNAs in the HS compared to the thermoneutral groups (Fig. 8D; Additional file 8B: Table S8B). Interaction networks between common experimentally validated target genes of the DE-EV-miRNAs and the organoids DEGs, are presented in Fig. 9A. Additionally, a focus on the DEGs involved in the cellular senescence pathway network and the DE-EV-miRNAs potentially targeting their function are represented in Fig. 9B.

**Table 4:** DE-miRNAs in oviductal organoid-derived EVs from the HS (42 °C) compared to the thermoneutral (37 °C) groups

**Upregulated miRNA**	**FC**	**FDR**	*FC* Fold Change, *FDR* False Discovery Rate **Downregulated miRNA**	**FC**	**FDR**
bta-miR-150	127.29	1.56E-21	bta-miR-221	-2.35	3.27E-02
bta-miR-92a	21.28	1.24E-13	bta-miR-11980	-4.13	7.36E-03
bta-miR-223	15.82	1.61E-03	bta-miR-23a	-4.26	1.04E-02
bta-miR-202	11.69	1.38E-05	bta-miR-370	-5.17	2.14E-04
bta-miR-16a	5.42	1.89E-02	bta-miR-6518	-10.17	5.05E-03
bta-miR-204	5.36	7.36E-03	bta-miR-197	-10.51	1.04E-02
bta-miR-181a	4.06	3.43E-03			
bta-miR-22-3p	3.96	1.04E-02			
bta-miR-9-5p	3.65	1.89E-02			
bta-miR-206	3.16	2.27E-02			
bta-miR-99a-5p	2.66	8.58E-04			
bta-miR-10b	2.62	7.36E-03			

*FC* Fold Change, *FDR* False Discovery Rate

### Quantitative real-time PCR validation of candidate genes and miRNAs

**Fig. 10:**
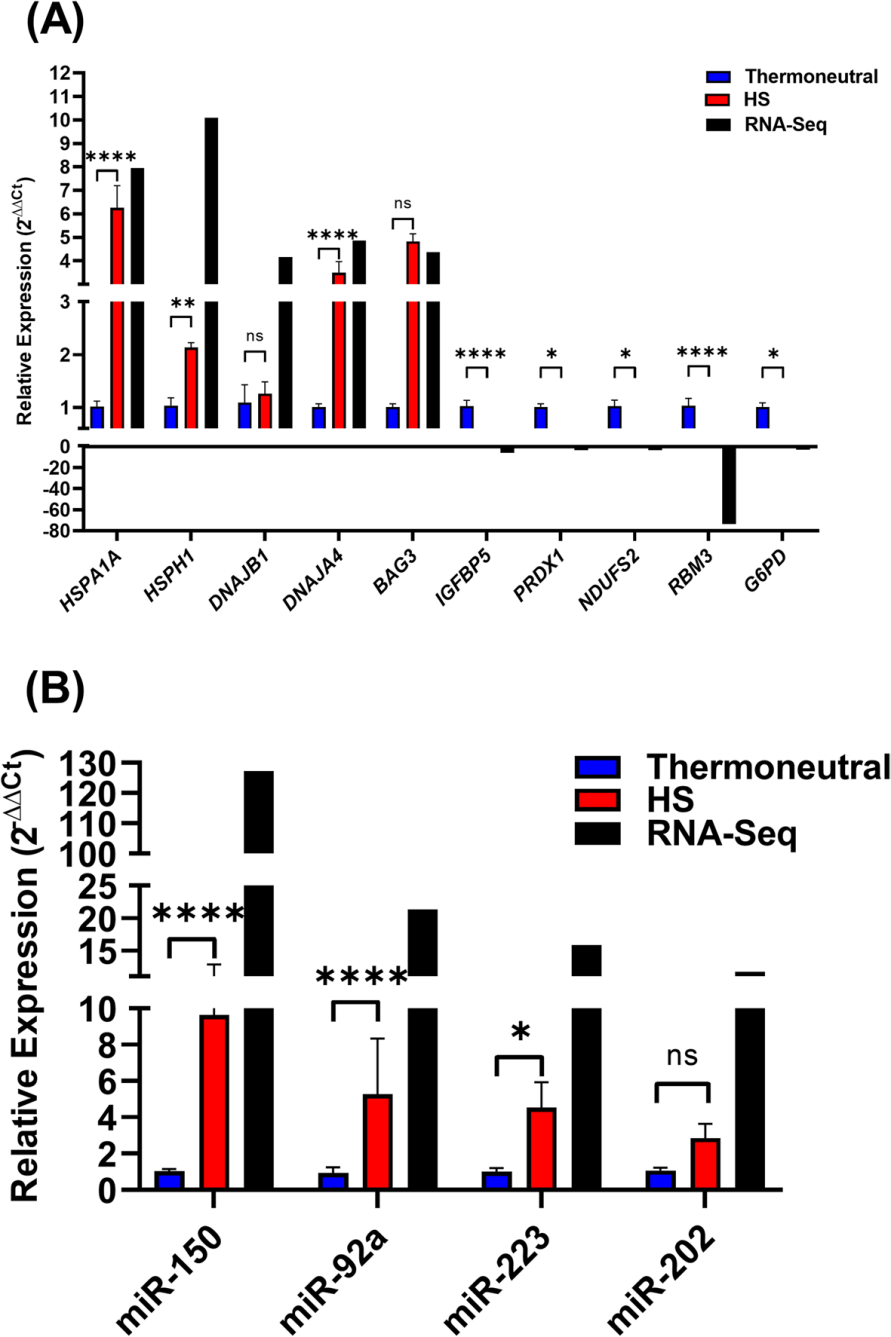
Quantitative Real Time PCR Validation. Expression validation of the selected DE-genes (**A**) and -miRNAs (**B**) compared against values calculated via RNA-seq analysis. Data are shown as the mean ± SEM and the differences between means were analyzed using one-way ANOVA followed by Tukey’s Multiple Comparisons Test in biologically independent samples (*n* = 4). Statistical significance (*) between the 42 °C compared to the 37 °C groups were determined at (*p* < 0.05)

To validate the sequencing data, 10 DE-genes (5 up- and 5 downregulated in the HS compared to the thermoneutral groups) and 4 upregulated miRNAs were selected for validation in 4 independent, biological replicates using quantitative real-time PCR. Selected candidate genes in oviductal organoids (Fig. 10A) and miRNAs in oviductal organoid-secreted EVs (Fig. 10B) show the same expression pattern consistent with the RNA-seq data.

### MicroRNA sequence motif and RBP identification

**Fig. 11:**
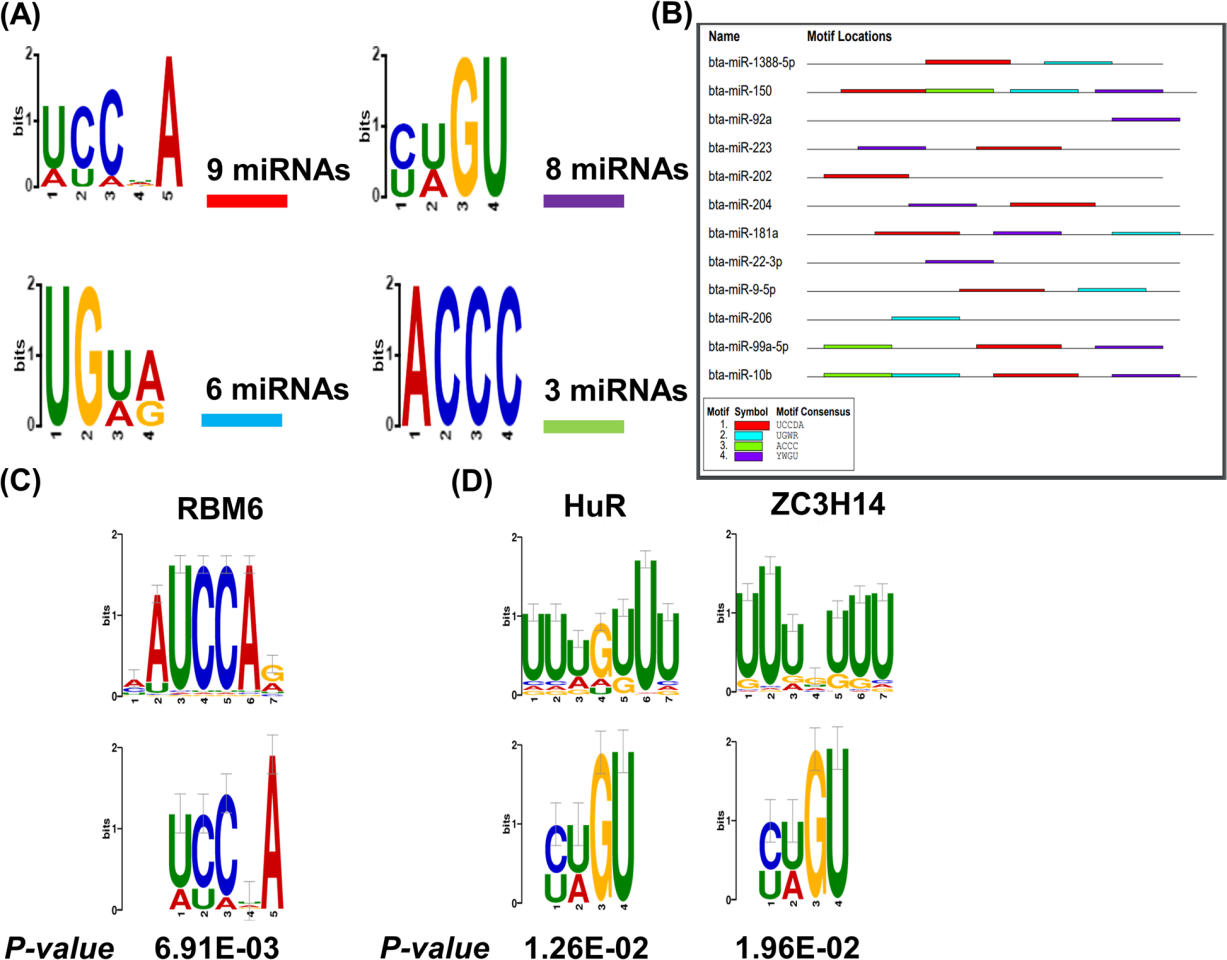
miRNA sequence motif analysis. Four identified, over-represented miRNA sequence motifs located amongst the upregulated miRNAs in differing numbers in the 42 °C compared to the 37 °C groups (**A**). Table depicting the location and distribution of the over-represented miRNA sequence motifs positioning on the upregulated miRNAs (**B**). Alignments of the ([U/A][C/U][C/A][U/G/A/C]A(**C**) and the [C/U][U/A]GU (**D**) miRNA sequence motifs with known RNA-binding proteins. (*p* < 0.05)

To investigate the potential mechanism of miRNA sorting and packaging into EVs by oviductal organoids in response to thermal stress, miRNA sequence motif analysis was performed for the miRNAs significantly enriched in EVs released in response to thermal stress using low abundant miRNAs (enriched in EVs released from organoids cultured under thermoneutral conditions) as controls. Accordingly, we have identified two miRNA motifs [U/A][C/U][C/A][U/G/A/C]A and [C/U][U/A]GU to be shared by 69% (9 of the 13) and 62% (8 out of 13) of the upregulated miRNAs, respectively (Fig. 11A). Moreover, an additional two motifs UG[U/A][A/G] and ACCC were identified and shared by 46% (6 of the 13) and 23% (3 out of 13) of the upregulated miRNAs, respectively. The sequence position and distribution of the identified motifs among the candidate miRNAs is indicated in Table 5 and Fig. 11B, respectively. Submission of the initial two significantly conserved motifs to the TOMTOM RBP identification tool showed that sequence motif ([U/A][C/U][C/A][U/G/A/C]A) is potentially targeted and bound by the RNA binding motif 6 (RBM6) protein (Fig. 11C), while sequence motif ([C/U][U/A]GU) is potentially targeted and bound by human antigen R (HuR) and zinc finger CysCysCysHis domain-containing protein 14 (ZC3H14) proteins (Fig. 11D).

**Table 5:**
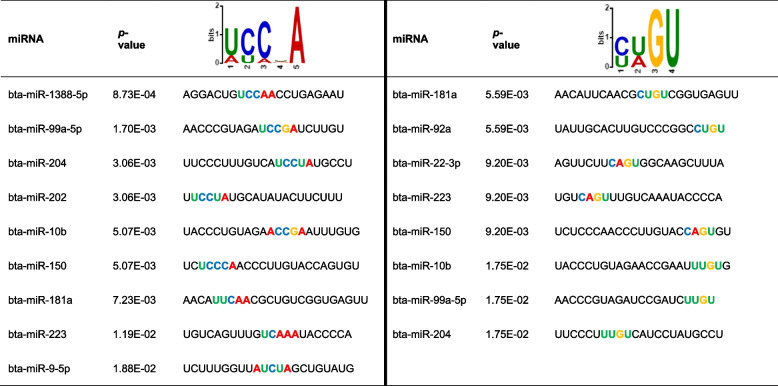
Identified motif positioning on the upregulated miRNAs in the HS (42 °C) compared to the thermoneutral (37 °C) groups

## Discussion

Oviductal fluid containing EVs have been identified as a major component safeguarding gametes from environmental stress [37, 38] and is regarded as the optimal milieu in which its composition reflects oviductal physiology [39]. Recent efforts have aimed to study the effects of HS on the oviduct both in vivo [40] and in vitro [41] through BOECs and EVs known to largely contribute and responsibly impact the composition of the oviductal fluid. In vivo, alterations in oviductal cells’ gene expression and EV concentrations have been reported, while similar significant alterations in *HSP70* and *OVGP1* expression were reported in vitro. *OVGP1* is a hormonally-regulated, carbohydrate-rich and traditionally-expressed protein-coding gene known to regulate receptivity and trophoblast adhesion. Alimiñana et al. [26], investigated the differential protein contents in BOECs and oviduct-derived EVs from in vivo and in vitro origin and revealed the absence of OVGP1 in in vitro cultured BOECs and corresponding EVs. Alternatively, in the present study oviductal organoids morphologically mirror their original tissue by forming round, cystic structures accompanied with an apical polarity in regard to a central lumen exhibiting encircling microvilli, consistent with previous reports of fallopian/oviductal organoids in mice [30, 31] and humans [29]. Moreover, our study revealed the detection of OVGP1, ZO1, and FOXJ1 in in vitro cultured organoids after passaging, indicating the suitability of our 3-D extended culture model to persist naïve-like identity, vital for the interpretation of the results in regard to oviductal physiology. Similarly, our gene panel revealed minimal differences amid in vitro cultured organoids in the mRNA expression of genes regulating cell proliferation and adhesion (*MKI67* and *CDH1*), ciliated cell function and differentiation (*LRRC6*, *TUBA1A*, *TUBA1B* and *TUBA1C*), and hormonal prostaglandin function (*PTGS2*, *PTGES* and *OXTR*) compared to naïve in vivo BOECs. Collectively, interspersed FOXJ1 + cells indicate ciliated cell presence, while stable *MKI67* mRNA levels amid in vitro cultured oviductal organoids and naïve in vivo BOECs reveal their active proliferation capacity. Similar to our previous work in endometrial organoids [42], in the present study ZO1 was found to be localized in both the nucleus and the cytoplasm. Such findings have been previously reported in monlayer cultures and several cell lines, and is posed to be correlated to the stage of cell growth and inversely connected to the extent and/or maturity of intercellular contacts [43]. Our study dually noted a significant increase in the transcript abundance for *TUBA1C*, which was also shown to be upregulated in oviductal fluid collected from the contra- versus ipsilateral bovine oviduct [44]. Additionally, in in vitro cultured HS oviductal organoids, the mRNA expression of *PTGS2* was signifncatly lower than in naïve in vivo BOECs, potentially indicating that the organoid cultures are not highly affected by in vitro ‘culture shock,’ which formally leads to an overproduction of reactive oxygen species (ROS) amidst cells [45], which has been shown in vivo [46] and in vitro [47] in regard to the oviduct. Additionally, in other studies the 3-D reproductive structures (organoids) have been shown to tolerably respond to exogenous hormonal stimuli [29, 48–50], a hallmark feature of mimicking in vivo function. In support of this notion, it was previously shown that small RNA profiles from 3-D culture-derived EVs of cervical cancer HeLa cells also showed to exhibit striking similarity (~ 96%) to circulating EVs found in vivo [51]. In this study, we have demonstrated for the first time the potential of oviductal organoids to systematically study the physiological and dynamic response of the oviduct to seasonal heat stress.

### HS-induced transcriptomic changes in oviductal organoids

High-throughput transcriptomic analysis of oviductal organoids cultured in vitro under thermoneutral and HS conditions revealed clear temperature-dependent clustering, indicative of a substantial physiological change governing oviductal organoids under HS conditions. In view of the oviduct and considering damaged cellular activity is prompted through HS (i.e. redox status and mitochondrial function, as well as protein folding), it is likely that the cellular response to the environment, acting as a dynamic machine, continuously diversifies and shifts an optimal milieu to a substandard microenvironment. These findings are in consonance with a recent in vivo study showing alterations in oviductal cells’ (ipsilateral and contralateral) gene expression from dairy cows under HS conditions [40]. Notably in our study, we found that of the 12,774 expressed genes, 11,725 genes were mutually expressed amongst both groups. Importantly, in terms of oviductal organoids long-term maintenance of naive-like physiology, in the present study, the RNA-sequencing dataset validated the presence of marker genes for BOEC function (*OVGP1*, *GPX4* and *HMGCR*) [52–55], which were stably expressed amid both groups of oviductal organoids following passaging during in vitro culture. Hence, the presented long-term 3-D organoid culture system functionally recapitulates certain validated aspects of the in vivo oviduct to satisfy novel descriptive studies, exemplified here, needed to address challenges in comprehending the early embryo–maternal interactions to conduct further functional studies.

Comparative analysis revealed, among the top highly abundant genes in both groups, we found genes related to early embryonic development (*ACTB* and *UBA52*), prostaglandin biosynthesis and the establishment/maintenance of pregnancy (*COX1*, *COX2* and *CST6*), as well as embryonic cellular senescence (*RPLP1* and *RPS15*). Uniquely, *COX1* is found to be upregulated as the transient embryo is present within the oviduct, suggesting synthesized prostaglandins interact with the embryo and facilitate embryo–maternal communication [56]. However, HS has been shown to largely disrupt the balance of vaginal prostaglandins (PGE2 and PGF2a), reducing oviductal smooth muscle motility, which is an important catalyst for the transportation of the gamete/embryo [57]. Correspondingly, in high abundance, were genes encoding functions in the sperm-oviduct interactions preceding early embryo development (*HSPA8* and *HSPA1A*), as well as genes reported to exert cryoprotective and anti-apoptotic effects as a consequence of stress (*HSPB1*). It is widely known that environmental cellular stress is directly connected to an immediate rapid induction of various heat shock proteins (HSPs). Convincing reports suggest HSPs (especially *HSPA1* and *HSPB1*), cohesively act as protective proteins through potentially downregulating signaling events leading to apoptosis [58]. Although, HSPs act as molecular chaperones critical for the maintenance of cellular homeostasis, increased amounts of HSPs can facilitate the suppression of overexpressed/mutated genes resulting from a loss of p53 function [59].

Upregulated genes (*CRISPLD2*, *HSPA6*, *SERPINE1*) in the thermal stressed groups could serve as potential indicators of the response of oviductal organoids to environmental heat stress. Higher expression of *CRISPLD2*, a secreted glycoprotein and target gene regulated by the P4-PR response, has been shown to potentially mediate the stromal-epithelial cross-talk during implantation and decidualization, largely dependent upon hormonal regulation [60]. Similarly, the HSP family member, *HSPA6*, shown to be involved in cellular response to stress, has been reported to be highly expressed in the ipsilateral oviduct likely in preparation for the presence of gametes and to facilitate embryo development [44]. Notably in our study, *SERPINE1*, which encodes a proteinase inhibitor, is shown to be involved in functional pathways governing cellular senescence. Cellular senescence contributes to the age-related decline in reproductive function and is known as a cellular response to stress aimed at inhibiting the proliferation of damaged cells, ultimately leading to a state of permanent growth arrest [61, 62]. Additionally, reports using *SERINE1* protease inhibitors to suppress oviductal protease activity through mediating the estrogen-epithelial ERa signaling, have been shown to be preeminent for fertilization and preimplantation embryo development [63].

In mammalian reproduction, it is widely known that mitochondria are inherited maternally and serve as energy factories, synthesizing the majority of the cells’ ATP predominantly through oxidative phosphorylation, in part, holding pertinent roles in metabolic functions governing embryonic development [64]. Results from the current study revealed the suppression of 1,348 genes in oviductal organoids following exposure to thermal stress, found to be involved in pathways related to oxidative phosphorylation and metabolic processes. Among the genes downregulated in oviductal organoids as a result of exposure to HS, *NDUFS8*, *NDUFS7*, *NDUFS6*, *NDUFS3*, *NDUFS2*, *COQ9*, *NDUFV2*, *ND3*, *NDUFV1*, *ND2* and *ATP5F1A*, *ATP5PB*, *ATP8*, *ATP6*, *ATP5MC2*, *ATP5MC3*, *ATP5F1C*, *ATP5F1D*, *ATP5MC1* are found to be involved in biological processes associated with mitochondrial function and ATP synthesis. Previous reports indicate that NADPH, supplies an antioxidant defense, while NADH promotes an increase in the oxidant load, generating ROS and sustained oxidative stress [65]. Therefore, genes suppressing the metabolic activity of high-functioning pathways (TCA cycle, pyruvate metabolism, fatty acid metabolism, etc.), promote an imbalance in mitochondrial NADH and NADPH, effecting ATP production, which leads to impairments in mitochondrial function, partly due to suppressed oxidative phosphorylation function. Interestingly, highly downregulated (FC = -73.4) *RBM3*, a stress-responsive gene, was shown to potentially have a mechanistic function to counteract the regulatory role of miRNAs by increasing inefficiencies in global protein synthesis under conditions of stress [66]. On the other hand, in agreeance with Stamperna and colleagues, our study concurred among the DEGs, the downregulation of *LOC512548* and *HP* (contralateral), as well as *SOCS3* (ipsilateral) in oviductal epithelial cells from dairy cows during the summer versus spring season [40]. Antileukoproteinase’s (*LOC512548)* role in inhibiting serine proteases, have been found to have antibiotic and anti-inflammatory functions in the human endometrium [67], as well as a coincidental function amidst the sperm-oocyte interaction in the human oviduct [68]. Taken together, our results demonstrate that the potential impact of seasonal heat stress on oviductal physiology is mediated by the dysregulation of genes responsible for mitochondrial function and energy metabolism, as well as reproductive and immune system responses.

### Oviductal organoid-derived EV-miRNA profiles in response to HS

Previous studies from our lab demonstrated the divergent EV-mediated molecular response of bovine granulosa cells to HS through their encapsulated miRNA cargoes, revealing their subsequent protective capacity against recurrent HS following co-incubation among naïve granulosa cells [14], as well as oocytes and embryos [15]. Similarly, in the present study, we detected a total of 251 EV-coupled miRNAs with 193 mutually expressed and 3 miRNAs (bta-miR-1246, bta-miR-148a, bta-miR-21-5p) sequentially expressed as the first, second and third most abundant among both groups. In vitro functional analysis in a previously reported study demonstrated the antiapoptotic measures of bta-miR-1246 by targeting *PCBP2* and *CREBL2* in HEK293 cells [69]. In accordance with others [70, 71], our study revealed a high prevalence of bta-miR-148a, which is found to target *DNMT1* and improve the developmental capacity of porcine somatic cell nuclear transfer embryos [72]. Furthermore, studies in mice showed that miR-21 targets *PTEN* [73], leading to the hypothesis that bta-miR-21-5p activity may activate AKT and ERK1/2 signaling pathways in the bovine corpus luteum to promote angiogenesis [74].

Specialized miRNAs packaged into EVs (23 exclusively expressed), reveal temperature-dependent variations in EV-encapsulated bioactive cargo resulting from oviductal organoids subjected to either HS or thermoneutral conditions. Among the miRNAs enriched in EVs released in response to HS, bta-miR-150, bta-miR-92a, bta-miR223 and bta-miR-202 were at minimum tenfold higher than in the thermoneutral groups. Pathway analysis of genes enriched by upregulated miRNAs include cellular senesce and p53 signaling pathways, which are critical in cell cycle processes involving proliferation and the maintenance of genomic and cellular integrity. Interestingly, in the present study, pathways related to cellular senescence are also the most significant pathways enriched by the DEGs in the oviductal organoids in response to HS. To our knowledge, this is the first evidence indicating bovine oviductal organoids use cellular senescence as a stress response mechanism to prevent stress-induced damage including oxidative stress, genotoxic damages and inflammation [75]. Notably, previous reports show that increased expression of miR-150 downregulates pro-apoptotic *P2X*_*7*_ mRNA expression in human cervical epithelial cancer cells [76]. Furthermore, as indicated in zebrafish, miR-92a-3p [77] and miR-202-5P [78], are maternally-derived miRNAs with critical functions in gametogenesis. It was shown that miR-92a-3p targets wee2, a cyclin-dependent kinase 1 inhibitor, regulating the cell cycle prior to the maternal-zygotic transition in cleavage-stage zebrafish embryos [77]. Additionally, miR-202-5P, which is enriched in EVs released in response to HS was also found to be highly abundant in early zebrafish embryos, suggesting its role in regulating primordial germ cell development throughout embryogenesis [78]. Therefore, although precautionary measures should be taken into account interpreting the species phylogenetic relationship, it is possible that oviductal organoids release EVs with the potential to shuttle specific miRNAs having a physiological role in early embryo development. Further functional studies to investigate the role of oviductal stress-associated EV-coupled miRNAs in regulating oviductal cells survival and potential to mediate early embryo development, is of great biological value.

In contrast to those miRNAs enriched in EVs released from oviductal organoids in response to HS, 35 miRNAs were found to be exclusively detected in EVs released from oviductal organoids cultured under thermoneutral conditions. Among the miRNAs downregulated in EVs from the HS groups compared to the thermoneutral groups were: bta-miR-197, bta-miR-6518, bta-miR-370, bta-miR-23a, bta-miR-11980 and bta-miR-221; with bta-miR-424-3P, bta-miR-654 and bta-miR-323 exclusively-differentially expressed in the EVs from thermoneutral cultured oviductal organoids. We found genes targeted by the downregulated miRNAs involve pathways regulating cellular senescence, as well as MAPK and FoxO signaling, with functions in cell-cycle arrest and intracellular signal transduction. Recent studies show miR-197-3p to be dramatically downregulated in ovarian tissues of patients with polycystic ovarian syndrome and in KGN human granulosa cell lines [79]. It was also confirmed that miR-197-3p targets *CUL3*, and its overexpression induces apoptosis by modulating caspase 3 and Bax (apoptotic) and bcl-2 (anti-apoptotic) protein expressions [79]. Similar reports indicate miR-370-3p inhibits cell proliferation and induces apoptosis in endometriotic cyst stromal cells by suppressing SF-1 [80]. These findings along with ours suggests the dysregulation of the aforementioned miRNAs due to HS might lead to the development of novel therapeutic targets underlying reproductive anomalies, organismal aging and human diseases.

We have previously shown the relevance of EV-coupled miRNA signaling in reproductive cells, in which certain miRNA species are selectively enriched in EVs in response to various stressors [14]. It seems clear that selective enrichment of EV-miRNAs stems in part from the physiological response of oviductal organoids to HS, with potential negative impacts on bystander cells and the gamete/embryo. However, detailed mechanisms underpinning how specific miRNAs are enriched in EVs released in response to HS are largely unknown. Recent reports indicated the involvement of specific RNA binding proteins (RBPs) and unique miRNA sequence motifs in EV-miRNA sorting versus cellular retention in a tissue/cell dependent manner [33–36]. To this point, in order to shed light on the potential mechanisms involved in miRNA sorting into EVs by oviductal organoids in response to HS, we employed computational technologies to identify miRNA sequence motifs and their target RBPs for 12 miRNAs enriched in EVs released from oviductal organoids following exposure to HS. Accordingly, two miRNA sequence motifs shared by 9 out of the 12 miRNAs were found in EV-coupled miRNAs released in response to HS. Further in silico analysis revealed that the identified motifs are recognized by RBPs, namely: RBM6, HuR and ZC3H14. Among these previous studies, it has been revealed that HuR, a regulator of cytoplasmic mRNA fate, alters the cellular response to proliferative, stress, apoptotic, differentiation, senescence, inflammatory and immune stimuli [81]. Moreover, cellular oxidative stress has implicated HuR to impair responses to oxidant damage that characterizes cellular senescence and advancing age [32]. Phosphorylated HuR using an ATP analog activated PKCα, promoted the export of HuR to the cytoplasm, where its target *COX2*, became more stable [32]. Notably, *COX1* and *COX2* play important roles in the smooth muscle layer and epithelial cells within the oviduct [56]. Regardless, further studies are required to investigate the interplay between miRNA motifs and RBPs in mediating the oviductal response to external stimuli, such as HS and their impacts on cellular survival and function during the maternal-embryo interaction.

## Conclusions

Our study incorporates both an innovative approach and novel techniques pushing the threshold to: 1) generate a new viewpoint on the oviducts’ capacity to undergo critical changes to external stimuli to safeguard the resulting gamete/embryo; 2) establish multifaceted, functional studies focused on oviduct signaling (organoids/EVs) and signatures potentially regulating stress response; 3) a potential platform to generate physiologically relevant EVs for implementation in current assisted reproductive technologies systems, aiming to enhance embryo survival and viability under various environmental and pathophysiological conditions (i.e. bystander effect). Finally, we have provided a comprehensive insight into the cellular and extracellular response of oviductal organoids to HS, yielding a structural footing to investigate the functional applicability and regulatory role of RNA cargoes (mRNA and miRNA) in modulating the fine-tuned oviductal microenvironment, essential for early stages of life. Further studies stemming from this work are needed to focus on the functional analysis of genes and EV-coupled miRNAs and their involvement in mediating the impact of physiological changes amid the oviduct microenvironment on the developing gamete/embryo and subsequent establishment of pregnancy. Taken together, oviductal organoids thusly provide a non-invasive approach to investigate normal and pathological reproductive processes, from a traditionally inaccessible organ.

## Methods

### Collection of bovine oviductal epithelial cells (BOECs)

Complete reproductive tracts including oviducts and ovaries (*n* = 10; a pool of 2 animals per replicate) were obtained from a local slaughterhouse (JBS Foods, USA) at the discretion of BPO Parts LLC (La Salle, CO) and transported within 1 h following collection via an insulated box containing physiological saline solution (0.9% NaCl) warmed to 37 °C to the Animal Reproduction and Biotechnology Laboratory for processing. Upon arrival, reproductive tracts were evaluated for the stage of the estrous cycle, dependent on the presence or absence of a fully formed, active corpus luteum with visible vasculature around its periphery. Resected ovaries containing complete contra- and ipsilateral oviducts from assessed stage II, diestrus tracts were initially washed through a physiological, warmed saline solution and subjected to a 70% ethanol rinse, followed by repetitive washings through a warmed saline solution. To offset the variability in abattoir-derived reproductive material, the same oviducts were represented in samples subjected to the differing ambient culture conditions (37 °C and 42 °C). Oviducts were trimmed from surrounding the tissues,opened longitudinally and processed according to previously defined measures [82, 83] by exposing the oviductal lumen, followed by scraping with a scalpel blade (Hill-Rom Holdings 371,120) in Handling Medium (HM) [HM; MEM Eagle with Earle's Salts (Sigma-Aldrich M2279) containing 25 mM HEPES (Sigma-Aldrich H0887), 100 U/ml Penicillin-0.1 mg/ml Streptomycin (Sigma-Aldrich P4333), 0.1 mM Pyruvate (Gibco 11,360–070), 2 mM Glutamax (Gibco 35,050–061) and Bovine Serum Albumin (5% v/v) (Sigma-Aldrich A9418)] to collect epithelial cells from the entire length of the oviduct for dissociation by enzymatic digestion. Subsequently, the solutions were passed through a 40 µm cell strainer (Greiner 542,040) to remove residual stromal and inflammatory cells then backwashed with RPMI-1640 medium (Gibco 11,875–085). The ensuing suspensions were then pelletized through centrifugation at 1000 xg for 10 min, washed in 1 mL DMEM/F-12 (Gibco 21,041–025), and finally resuspended in 20x (v/v) phenol red-free, growth factor reduced UltiMatrix (Cultrex; Boitechne BME001-05).

### Oviductal organoid culture

Parameters to establish organoid cultures were determined as we have previously described [50]. Unless indicated otherwise, all chemicals were manufactured by Sigma-Aldrich. Briefly, the homogenized UltiMatrix suspension, as described above, was aliquoted in 25 µL microdrops in a 48-well culture plate (Corning 3548), and allowed to strengthen formation for 30 min at 37 °C. The 3-D suspensions were then overlayed with 250 µL of Organoid Medium (OM) (OM; DMEM/F-12 without phenol red (Gibco 21,041–025), 100 μg/mL primocin (Invivogen ant-pm-1), 2.5 mM L-glutamine, 2% B27 Plus (Gibco A35828-01), 1% N2 (Gibco 17,502–048), 1% Insulin–transferrin–selenium (Gibco 41,400–045), 1 mM nicotinamide (N0636), 50 ng/mL recombinant human EGF (R&D Systems 236-EG), 50 ng/mL recombinant human FGF-10 (PeproTech 100–26), 100 ng/mL recombinant human Noggin (R&D systems 6057-NG/CF), 0.5 μM TGFβ/Alk inhibitor A83-01 (Tocris 2939), 1.25 mM N-acetyl-L-cysteine (EMD Millipore 106,425), 10 μM SB202190 (S7067), 10 μM Y27632 (EMD Millipore 688,000) as previously described [50, 82, 83]. For all experiments, organoid culture suspensions were in a confined chamber regulated to 37 °C and 5% CO_2_ in humidified air for growth and development prior to exposure to 42 °C to induce thermal stress. The OM was refreshed by half (v/v) every two to three days with passaging occurring weekly every 7^th^ day. For passaging, the UltiMatrix containing the organoids were collected and pelletized via centrifugation at 1,000 xg for 10 min, washed in 1 mL of DMEM/F-12 with agitation applied from vigorous pipetting to induce dissociation and yield mostly single cells, followed by replating according to the same procedure described above. Organoids of low passage number (P1D7) were used for the experiments described.

### Histology

Histological preparation of intact oviduct and oviductal organoids was performed as we have previously described [82, 83]. Briefly, organoid VitroGel^®^ suspensions were soaked in 250 µL of VitroGel^®^ Cell Recovery Solution and washed with DMEM/F-12 followed by DPBS (1X) and centrifuged at 600 xg for 6 min. After centrifugation, organoid pellets were fixed in 4% paraformaldehyde for 30 min and embedded in 2% agarose gel (Bio-Rad 1,613,101). Agarose-embedded organoids were stored in 70% ethanol until processing, then embedded in paraffin wax, sectioned (6 μm), and stained using hematoxylin and eosin (H&E) and assessed for normal oviductal epithelial cell architecture.

### Immunohistochemistry

Organoid preparation for immunohistochemistry was performed as we have previously described [50, 82, 83]. Briefly, paraffin wax embedded organoids were mounted on Superfrost Plus slides, deparaffinized then rehydrated through graded alcohols until distilled water. Antigen retrieval was then accomplished by subjection to citrate buffer (pH 6; 96 °C for 20 min) and blocked with hydrogen peroxide then washed in distilled water. Slides were then incubated with primary (4 °C overnight) and HRP-conjugated and/or biotinylated secondary antibodies (OVGP1; Biotin Ms IgG, ZO1; Rb IgG HRP, and FOXJ1; Biotin Rb IgG), respectively. A list of the antibodies and dilutions is indicated in (Additional file 9: Table S9). Negative reagent controls provided utilized the same staining procedures in the absence of the primary antobody incubation step. Prior to microscopy evaluation, slides were activated using the RTU Vectastain kit (Vector Laboratories) for 30 min and visualized using Vectastain ABC-HRP and DAB substrate solution (Vector Laboratories) counterstained with hematoxylin. Incubations took place at a room temperature environment with TBS-T solution used as a washing buffer. Slides were assessed for localization and presence of OVGP1, ZO1, and FOXJ1 staining.

### Exposure of oviductal organoids to heat stress

Following the culmination of the first passage (14 days from initial collection), organoids were removed from UltiMatrix using Organoid Harvesting Solution (Cultrex; Biotechne 3700–100-01) and transferred to VitroGel^®^ (TheWell Bioscience VHM04-K), a synthetic, xeno-free extracellular matrix. At this point, the 3-D matrices were incubated for 30 min at 37 °C to become less fluidic, according to the procedure described above. Finally, the OM was replenished followed by culturing for an initial 24 h priming period at 37 °C and 5% CO_2_ in humidified air. Thermoneutral organoids remained at 37 °C for an additional 24 h, while for experiments with heat stress organoids were transferred to a confined chamber regulated to 42 °C in an atmosphere of 5% CO_2_ in humidified air for the remaining 24 h. Experiments were replicated a total of eight times to gauge variability in abattoir-derived reproductive material for both culture environments.

### Collection of cultured oviductal organoids and conditioned medium for downstream analysis

Following the collection of conditioned OM, the 3-D organoid suspensions were collected and suspended in VitroGel^®^ Cell Recovery Solution (TheWell Bioscience MS03-100), washed with DMEM/F-12 and pelletized by centrifugation at 600 xg for 6 min. Organoid pellets were snap frozen in liquid nitrogen and stored at -80 °C for downstream use. The EV-containing conditioned OM were then centrifuged in sequential order at 500 xg for 10 min, 3,000 xg for 10 min and 17,200 xg for 30 min and filtered through a 0.22 µm sterile filter (Millex^®^ SLGPR33RB) to remove residual cellular contaminants, large particles and protein aggregates. All centrifugation steps were performed at 4 °C. Bovine oviductal organoid-derived EVs were isolated from ~ 10 mL of conditioned OM (8 replicates/treatment group) using ultracentrifugation followed by size exclusion chromatography (SEC). Briefly, OM were subjected to two consecutive rounds of ultracentrifugation at 120,000 xg for 70 min in the Optima XE-90 Ultracentrifuge using the SW 55Ti rotor (Beckman Coulter A94471). Isolated EVs were further standardized by elution in 180 µL of sterile DPBS (1X) (ThermoFisher Scientific 14,190–144) using the Exo-spin™ mini columns (CELL guidance systems EX03-50) according to the manufacturer’s protocol. Isolated EVs were then stored at -80 °C for further characterization and molecular analysis.

### Characterization and quantification of oviductal organoid-derived EVs

EV identification and characterization were carried out according to guidelines detailed by the International Society for Extracellular Vesicles [84], including EV marker protein validation, nanoparticle concentration/size distribution and morphological visualization as described below. Detailed information regarding experimental procedures has been submitted to the EV-TRACK knowledgebase (https://evtrack.org/) (EVTRACK ID: EV230000)[85].

#### Western blotting (WB)

Immunoblotting technique was performed for isolated EVs to detect EV and cellular protein markers. For this, proteins were extracted from oviductal organoid cells and the corresponding EVs using RIPA buffer (Marker Gene™ Technologies M2777). Protein lysates were then boiled with 4 × Laemmli Sample Buffer (Bio-Rad 1,610,747) and 2-Mercaptoethanol (Bio-Rad 1,610,710) at 95 °C for 5 min prior to loading on a 4–15% (w/v) Tris–Glycine Stain Free Gel (Bio-Rad 4,568,084). Electrophoresis was performed for 15 min at 90 V followed by 30 min at 200 V with Precision Plus Protein Dual Color Standards as a molecular weight marker (Bio-Rad 1,610,374). Separated proteins were transferred (100 V for 60 min at 4 °C) to a 0.20-µm pore nitrocellulose membrane (Bio-Rad 1,620,112) and blocked for 1 h at room temperature in 5% (w/v) Non-Fat Dry Milk (Bio-Rad 1,706,404) and further incubated over night at 4 °C with a primary antibody against each marker protein (System Biosciences; rabbit polyclonal TSG101 EXOAB-TSG101-1, rabbit polyclonal FLOT1 EXOAB-FLOT1-1, rabbit polyclonal CD63 EXOAB-CD63A-1) (Sino Biological rabbit polyclonal Cytochrome C 102139-T42). Afterwards, membranes were washed 5 times with TBS-T solution and exposed to the secondary antibody (System Biosciences; Goat anti Rabbit HRP) for 1 h at room temperature. Bands and signals were enhanced using SuperSignal™ West Pico PLUS Chemiluminescent Substrate (ThermoFisher Scientific 34,579) and imaged using the ChemiDoc XRS + chemiluminescence system with Image Lab™ Software (version 6.0.1 build 34) (Bio-Rad). A list of the antibodies and dilutions is indicated in (Additional file 9: Table S9) with full length blots provided (Additional file 10: Fig. S1).

#### Nanoparticle tracking analysis (NTA)

A ZetaView^®^ QUATT 4 Nanosight Instrument (Particle Metrix) was used to determine the particle concentration and size distribution in all EV samples. Briefly, isolated EVs were diluted in sterile DPBS (1X) and loaded into the device and analyzed. Measurements were taken at 11 positions via scatter mode using a 488 nm laser to determine the size distribution and concentration within a sample. Analysis of EV concentrations were calculated using the Zetaview Software (version 8.05.12 SP1) before and after sample dilution.

#### Transmission electron microscopy (TEM)

For morphological evaluation of oviductal organoid-derived EVs, we performed negative staining TEM of EVs by adsorption onto mesh copper grids with carbon-coated formvar film (Electron Microscopy Sciences FCF 400-Cu). For this, grids were placed film side down on parafilm containing drops of 1% Alcinan Blue 8GX (Sigma-Aldrich 33,864–99-2) for 5 min, followed by washing with ddH_2_O for 15 s. Afterwards, the grids were floated on a 5 µL drop of isolated EVs for 10 min, prior to fixing in 2.4% uranyl acetate (Electron Microscopy Sciences 22,400) for 30 s. After fixing, samples were set to dry for imaging on a JEOL JEM-1200 EX transmission electron microscope.

### RNA isolation

Total RNA was isolated from organoid cells (5 replicates/treatment group, a pool of samples from 2 animals per replicate) using an RNeasy^®^ Plus Micro Kit (Qiagen 74,034). To remove genomic DNA contaminants, on-column DNA digestion was performed using RNase-free DNase (Qiagen; Hilden, Germany). Similarly, an Exosomal RNA Isolation Kit (Norgen Biotek Corp. 58,000) was used to isolate total RNA enriched with small RNA from oviductal organoid-derived EVs (3 replicates/treatment group, a pool of 2 animals per replicate) according to the manufacturer’s protocol. In both cases, RNA quality control and concentration were assessed using an Agilent 2100 Bioanalyzer (Agilent Technologies G2939BA) and NanoDrop™ 2000 Spectrophotometer (ThermoFisher Scientific ND-2000), respectively.

### RNA library preparation and sequencing

RNA libraries and next-generation sequencing (NGS) were conducted by Novogene Co., LTD (California, USA). For mRNA transcriptome profiling, RNA libraries were generated using a NEBNext^®^ Ultra™ RNA Library Prep Kit for Illumina (NEB E7530L) and for miRNA profiling, small-RNA libraries were prepared using a TruSeq Small RNA Library Prep Kit (Illumina RS-200) according to the manufacturer’s instructions. Library quantity and quality assessments were performed using a Qubit^®^ DNA HS Assay Kit on a Qubit^®^ 2.0 Fluorometer and an Agilent DNA High Sensitivity Kit in an Agilent 2100 Bioanalyzer, respectively. Library concentrations were calculated using quantitative PCR measures then pooled in equimolar ratios and sequenced in a NovaSeq6000 sequencing instrument (Illumina) as paired (150 bases) or single-end (50 bases) reads for the transcriptome and miRNA sequencing, respectively.

### RNA-seq data analysis

FASTQ files were generated for each sample using the software bcl2fastq (Illumina) with FastQC tool version 0.11.9 assessing their quality. Data were analyzed using the CLC Genomics Workbench software, version 21 (www.qiagenbioinformatics.com). Trimming of raw sequencing reads was based on quality score (Q‐score > 30), ambiguous nucleotides (maximum two nucleotides allowed), read length (≥ 15 nucleotides), and removal of adapter sequences. Reads were mapped to the bovine reference genome (ARS-UCD1.2) and small-RNA reads were annotated against bovine precursor and mature miRNAs listed in the mirBase database (release 22) using the CLC Genomics Workbench RNA-seq Analysis and Quantify miRNA tools, respectively, with default software parameters applied. Normalization of raw expression data was performed using the trimmed mean of M-values (TMM) normalization method [86] and presented as either transcripts per million (TPM) or TMM-adjusted counts per million (CPM), for genes and miRNAs, respectively. For genes, the expression threshold was determined by the zFPKM method using the zFPKM R package v.1.16.0 [87]. The zFPKM transform/normalization method is used to determine, with high consistency, the threshold of expressed transcripts avoiding the low-abundance and biological noise transcripts due to sequencing errors, and/or off-target read mapping. zFPKM values are calculated from the distribution of the log2 (TPM values) after fitting to the right side of the major peaks of each gene expression curve to a half-Gaussian curve, mirrored into a full Gaussian distribution, and then transformed log2(TPM) into zFPKM from this fit. Based on the recommended zFPKM score that considers the expressed/active genes [79], genes with zFPKM >  − 3 in all replicates were considered as expressed. A miRNA with an average value of CPM ≥ 10 was considered to be expressed. The CLC Genomics Workbench Differential Expression tool was used for the expression analysis comparison of the two groups. Differential expression was considered based on a negative binomial Generalized Linear Model (GLM) function. DEGs and DE-miRNAs were filtered based on FC ≥ 2 and *P-*adjusted value FDR < 0.05 [88]. The raw FASTQ and processed CSV files for the transcriptome and miRNA sequencing data have been deposited in the NCBI’s Gene Expression Omnibus (GEO) (https://www.ncbi.nlm.nih.gov/geo/) with the accession numbers GSE221929 and GSE221895, respectively.

### Target gene prediction, ontological classification and pathway analysis

Genes targeted by the DE-miRNAs were identified using the miRWalk database [89]. Within miRWalk, validated target genes from miRTarBase (version 7.0) and commonly targeted genes predicted by TargetScan (version 7.1) and miRDB (release 5.0) were selected for ontological classification and pathway analysis. Both targeted genes and DEG lists were submitted to the Database for Annotation, Visualization, and Integrated Discovery (DAVID) Bioinformatics web tool v. 2021 (http://david.abcc.ncifcrf.gov/) for pathways and ontological classification enrichment analysis using the EASE Score (a modified Fisher Exact p-value) with a threshold of less than or equal to 0.05. Pathways and biological processes (BPs) were determined from the KEGG database [90], and GOTERM_BP_DIRECT annotation set, respectively. The interaction networks of the miRNAs, targeted genes, and DEGs were constructed with Cytoscape software [91].

### Quantitative real time PCR validation of candidate mRNA and miRNA

In order to validate the in vivo biomimicry of in vitro produced organoids, as well as the NGS data for DEGs and DE-miRNAs, quantitative real-time PCR (qRT-PCR) expression analysis was performed for selected transcripts and miRNAs in oviductal organoid cells and oviductal organoid-derived EVs, respectively. Additionally, naïve BOEC samples were used as a control in the organoid characterization experiment. For the functional characterization of in vitro produced organoids and validation of candidate DEGs, equal amounts of total RNA (~ 638 ng/µL and ~ 1024 ng/µL, respectively) extracted from independent organoid samples was reverse transcribed to generate cDNA, using the SuperScript™ III First-Strand Synthesis Super Mix Kit (Invitrogen 18,080,400) with random hexamer and oligo d(T) 20 primers, according to the manufacturer’s instructions. The gene panel were strategically constructed to characterize the functionality of in vitro produced organoids (*n* = 3), while ten candidate genes for the validation of the DEGs (*n *= 4) were randomly selected, with gene-specific primers designed using the Primer-Blast tool (https://www.ncbi.nlm.nih.gov/tools/primer-blast/). All primers are indicated in Additional file 11: Table S10. Quantification of each candidate gene was performed in 20 µl reaction volumes containing 0.5 μL of each primer (10 µM), 10 μL of iQ SYBR Green Supermix (Bio-Rad 1,708,880), 7 μL of ddH_2_O and 2 μL of the cDNA template. The following thermocycling conditions were applied for amplification: initial denaturation at 95 °C for 3 min, followed by 40 cycles of amplification at 95 °C for 15 s., 60 °C for 30 s., 72 °C for 30 s. The specificity of the amplification was determined from the melting curve analysis generated following each run. The geometric mean of expression levels of *β-ACTIN* and *GAPDH* was used to normalize the expression of the candidate genes.

Four DE-miRNAs (bta-miR-150, bta-miR-92a, bta-miR-223 and bta-miR-202) were chosen for quantification in the samples described above using TaqMan™ miRNA Assays (Applied Biosystems™) according to the manufacturer's instructions. Briefly, 5 ng/µL of total RNA were reverse-transcribed using a TaqMan™ microRNA Reverse Transcription Kit (Applied Biosystems™ 4,366,596). The reaction mixture (7.5 μL) constituted 0.75 μL dNTP mix w/dTTp (100 M total), 0.075 μL 10X RT buffer, 0.095 μL RNase inhibitor (20U/μL), 0.5 μL Multiscribe™ RT enzyme (50U/μL), 1.5 μL of the given miRNA-specific stem-loop primer, and 2.5 μL of RNA (5 ng/μL). The miRNA-specific cDNA reaction mixture was incubated at 16 °C for 30 min, 42 °C for 30 min then followed by 85 °C for 5 min For miRNA quantification, 20 μL qRT-PCR mixtures were prepared in a 96-well PCR plate with 10 μL TaqMan™ Universal PCR Master Mix, no AmpErase™ UNG (Applied Biosystems™ 4,324,018), 1 μL miRNA-specific TaqMan microRNA probe (FAM, 250 nM), 7 μL ddH_2_O and 2 μL of the RT product. The following thermocycling conditions were applied for amplification: initial denaturation at 95 °C for 10 min, followed by 40 cycles of amplification at 95 °C for 15 s. and 60 °C for 1 min Expression normalization was performed against small nuclear RNA (U6), as it stably resembled the mean expression values for all miRNAs. All qRT-PCR validation analyses were performed using the CFX96 Touch Real-Time PCR Detection System (Bio-Rad) and expression data analysis was performed using a comparative threshold cycle (CT) method [92]. All data are shown as the mean ± SEM and the differences between means were analyzed using one-way ANOVA followed by Tukey’s Multiple Comparisons Test in biologically independent samples (*n* = 4). Statistical significance (*) between the 42 °C compared to the 37 °C groups were determined at (*p* < 0.05).

### MicroRNA sequence motif and RBP identification and analysis

Sequence-specific miRNA motifs (4–6 nucleotides), enriched in the upregulated miRNAs (including the significantly, exclusively expressed miRNAs; *n* = 13) versus the downregulated miRNAs (including the significantly, exclusively expressed miRNAs; *n* = 9 as control sequences) in EVs derived from oviductal organoids exposed to HS, were identified using Multiple Expectation Maximization for Motif Elicitation (MEME) suite v. 5.5.0 https://meme-suite.org [93]. Motifs commonly detected in at least 50% of the submitted miRNAs were selected for further analysis to determine the motif-associated RBPs using a TOMTOM motif comparison tool [94].

### Supplementary Information

**Additional file 1: Table S1. **Summary of sequence reads for oviductal organoids mapped to the bovine reference genome.

**Additional file 2: Table S2. **Differentially expressed genes in 42°C compared to 37°C group.

**Additional file 3: Table S3A. **KEGG pathway analysis for differentially expressed genes enriched in 42°C compared to 37°C group. **Table S3B. K**EGG pathway analysisfor differentially expressed genes enriched in 42°C compared to 37°C group.

**Additional file 4: Table S4A. **Biological process (BP) analysis for differentially expressed genes enriched in 42°C compared to 37°C group. **Table S4B. **Biological process (BP) analysis for differentially expressed genes enriched in 42°C compared to 37°C group.

**Additional file 5: Table S5. **Summary of sequence reads for oviductal organoid EVs mapped to the bovine reference genome and annotated against bovine miRNAs listed in the mirBase database.

**Additional file 6: Table S6. **A complete list of all expressed miRNAs indicated as the mean of TMM-adjusted Counts Per Million (CPM) value.

**Additional file 7: Table S7A. **KEGG pathway analysis for genes targted by the differentially expressed miRNAs in 42°C compared to 37°C group. **Table S7B. **KEGG pathway analysis for genes targted by the differentially expressed miRNAs in 42°C compared to 37°C group.

**Additional file 8: Table S8A. **Biological process (BP) analysis for genes targted by the differentially expressed miRNAs in 42°C compared to 37°C group. **Table S8B. **Biological process (BP) analysis for genes targted by the differentially expressed miRNAs in 42°C compared to 37°C group.

**Additional file 9: Table S9. **List of Antibodies used.

**Additional file 10: Figure S1. **Western blot analysis of extracellular vesicle and  cellular protein markers.

**Additional file 11: Table S10. **Sequence specific primers used for qRT-PCR analysis.

## Data Availability

Datasets generated during the current study including the raw FASTQ files and processed CSV files for genes and miRNAs were deposited in the National Center for Biotechnology Information (NCBI) Gene Expression Omnibus (GEO) database (https://www.ncbi.nlm.nih.gov/geo/) and can be accessed with accession numbers GSE221929 and GSE221895, respectively. Experimental procedures related to EV experiments have been submitted to the EV-TRACK knowledgebase (https://evtrack.org/) (EVTRACK ID: EV230000).

## Abbreviations

HS: Heat stress
EV: Extracellular vesicle
BOEC: Bovine oviductal epithelial cell
HSP: Heat shock protein
ROS: Reactive oxygen species
RBP: RNA-binding protein
HM: Handling medium
OM: Organoid medium
SEC: Size exclusion chromatography
NTA: Nanoparticle tracking analysis
TEM: Transmission electron microscopy
FC: Fold change
QC: Quality control
NGS: Next-generation sequencing
PCA: Principal component analysis
GO: Gene ontology
TMM: Trimmed mean of M-values
TPM: Transcripts per million
CPM: Counts per million
GLM: Generalized linear model
DEG: Differentially expressed genes
DE-miRNAs: Differentially expressed miRNAs
GEO: Gene expression omnibus
DAVID: Database for annotation, visualization, and integrated discovery
BP: Biological processes
MEME: Multiple expectation maximization for motif elicitation
NCBI: National center for biotechnology information
