# Supplementary material for: Bovine oviductal organoids: a multi-omics approach to capture the cellular and extracellular molecular response of the oviduct to heat stress
Source: BMC Genomics. 2023 Oct 27;24:646. doi: 10.1186/s12864-023-09746-y (PMC10605953; doi:10.1186/s12864-023-09746-y)
Supplement: Supplementary file 9 — Additional file 9: Table S9. List of Antibodies used. [file 12864_2023_9746_MOESM9_ESM.docx]

| **Target** | **Catalog #** | **Dilution** | **Manufacturer** |
| --- | --- | --- | --- |
| FOXJ1 | HPA005714 | 1:100 | Sigma Aldrich |
| ZO1 | 61-7300 | 1:200 | Invitrogen |
| OVGP1 | sc-377267 | 1:50 | Santa Cruz Biotechnology |
| SignalStain® Boost IHC Detection Reagent (HRP, Rabbit) | #8114 | NEAT | Cell Signaling Technologies |
| Goat Anti-Rabbit IgG Antibody (H+L), Biotinylated | BA-1000 | 1:500 | Vector Laboratories |
| Horse Anti-Mouse IgG Antibody (H+L), Biotinylated | BA-2000 | 1:500 | Vector Laboratories |
| Anti-TSG101 | EXOAB-TSG101-1 | 1:100 | System Biosciences |
| Anti-FLOT1 | EXOAB-FLOT1-1 | 1:100 | System Biosciences |
| Anti-CD63 | EXOAB-CD63A-1 | 1:100 | System Biosciences |
| Cytochrome c | 102139-T42 | 1:200 | Sino Biological |
| Additional File Table S9. List of Antibodies used. |  |  |  |
