# Supplementary figures and images for: Bovine oviductal organoids: a multi-omics approach to capture the cellular and extracellular molecular response of the oviduct to heat stress
Source: BMC Genomics. 2023 Oct 27;24:646. doi: 10.1186/s12864-023-09746-y (PMC10605953; doi:10.1186/s12864-023-09746-y)

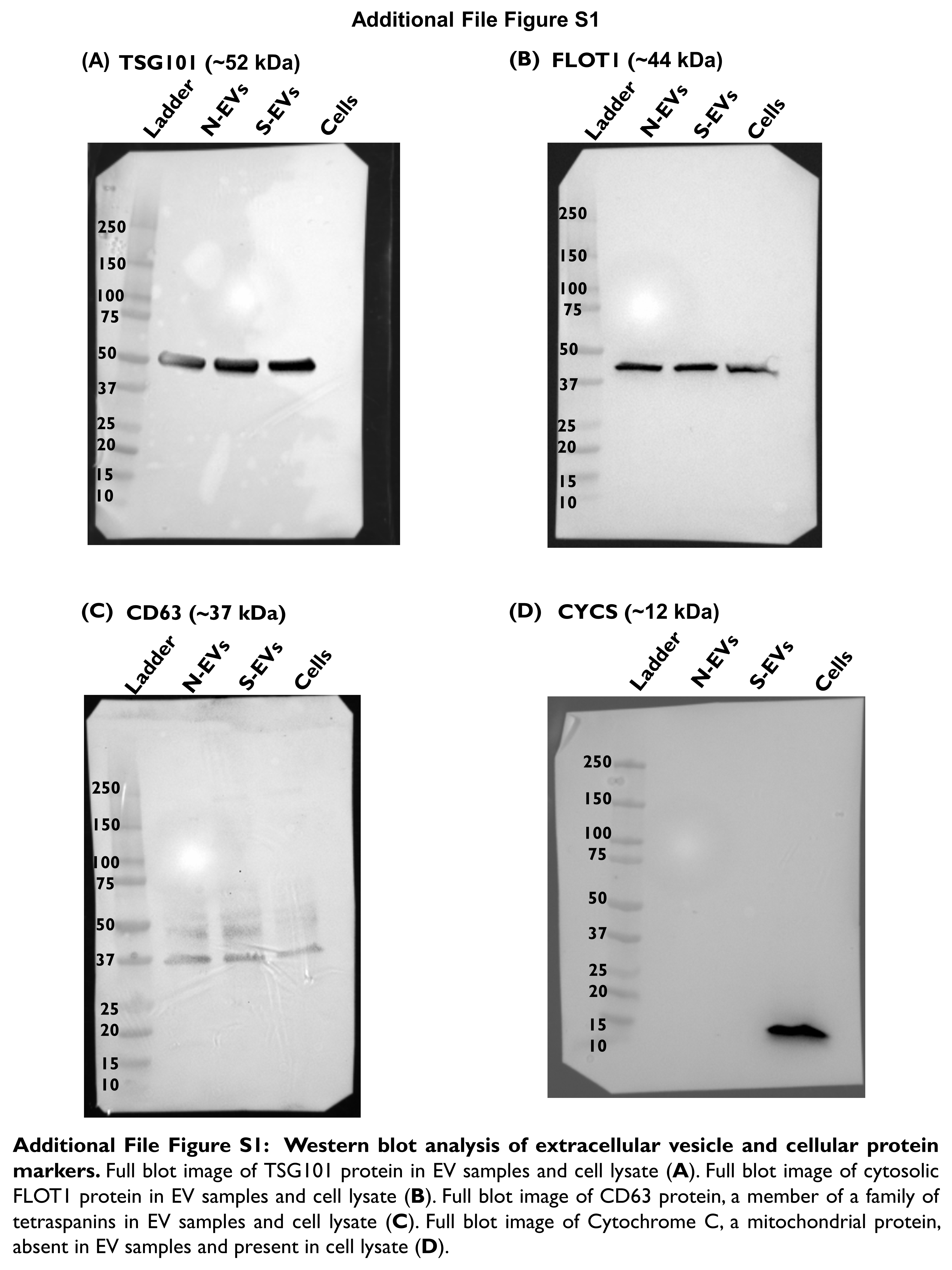

Supplement: Supplementary file 10 — Additional file 10: Figure S1. Western blot analysis of extracellular vesicle and cellular protein markers. [file 12864_2023_9746_MOESM10_ESM.tif]
