# Supplementary material for: Bovine oviductal organoids: a multi-omics approach to capture the cellular and extracellular molecular response of the oviduct to heat stress
Source: BMC Genomics. 2023 Oct 27;24:646. doi: 10.1186/s12864-023-09746-y (PMC10605953; doi:10.1186/s12864-023-09746-y)
Supplement: Supplementary file 11 — Additional file 11: Table S10. Sequence specific primers used for qRT-PCR analysis. [file 12864_2023_9746_MOESM11_ESM.docx]

| **Gene Name** | **Accession Number** | **Primer Sequences** |
| --- | --- | --- |
| MKI67 | XM_015460791.2 | F5`-GCAGCTTCGGTGATTCCACG-3´ R5´-CAGGCAACCCCCAAAGGAGA-3´ |
| CDH1 | NM_001002763.1 | F5`-GGGCTGGACCGTGAGAGTTTT-3´ R5´-GTTGTGCTCAAGCCTTCGCC-3´ |
| LRRC6 | XM_005207958.4 | F5`-TGCAATGAGTCCATAGGCAAGGA-3´ R5´-GGAAAAGCGCACTTCCTGGG-3´ |
| TUBA1A | NM_001166505.1 | F5`-CATCCCCATGGTGACCGAGTT-3´ R5´-GAGATGCACTCACGCATAGTTG-3´ |
| TUBA1B | NM_001114856.1 | F5`-AACCGGGACCCGTGTCTACT-3´ R5´-GGAGATGCACTCACGCATGG-3´ |
| TUBA1C | NM_001034204.1 | F5`-ACTTCTCCCCCGGACTCCTTA-3´ R5´-AGATGCACTCACGCATAACGG-3´ |
| PTGS2 | NM_174445 | F5`-CGATGAGCAGTTGTTCCAGA-3´ R5´-GAAAGACGTCAGGCAGAAGG-3´ |
| PTGES | NM_174443.2 | F5`-GGCTGCGGAAGAAGGCTTTTG-3´ R5´-TCCACATCTGGGTCGTTCCG-3´ |
| OXTR | NM_174134.2 | F5`-GCGCCCAAGGAAGCCTCAC-3´ R5´-AGGAAGCGCTGCACAAGTTC-3´ |
| HSPA1A | NM_203322 | F5`-GGGGAGGACTTCGACAACAG-3´ R5´-GAAGTCGATGCCCTCGAACA-3´ |
| HSPH1 | NM_001075302 | F5`-TCCCAGATGCTGACAAAGCTA-3´ R5´-CAGCTTGTCCCTGAACTCGT-3´ |
| DNAJB1 | NM_001033763 | F5`-CGTCGGACGAGGAGATCAAG-3´ R5´-CACTGCCCTTCAGGCCTTC-3´ |
| DNAJA4 | NM_001102120 | F5´-CCCTTCAGGCCACCCGAT-3´ R5´-CTTCACCATCTTGTCTCCGGTAT-3´ |
| BAG3 | NM_001082471 | F5´-ATCAAGATCGACCCGCAGAC-3´ R5´-AGAGGATGCGGTTTCCTTGG-3´ |
| IGFBP5 | NM_001105327 | F5´-GCAAGCCAAGATCGAAAGAGAC-3´ R5´-GGGCCCCTGCTCAGATTTC-3´ |
| PRDX1 | NM_174431 | F5´-TCCTATTTCAGTGGAACTGCTGAT-3´ R5´-AAGCAATGATCTCCGTGGGG-3´ |
| NDUFS2 | NM_001075137 | F5´-CACCGGGGCACTGAGAAG-3´ R5´-GTGATTTCTCCAAATAGCACTCGG-3´ |
| RBM3 | NM_001303463 NM_001303480 | F5´-TGACAACTGAGATGAGAAACACA-3´ R5´-ACAAAACAAACATGGAGCTTTCT-3´ |
| G6PD | NM_001244135 | F5´-GAGCAGCGAAGCACAGAGAG-3´ R5´-GCAAAAGGCCATCTCGGAAC-3´ |
| β-ACTIN | NM_173979 | F5´- TGTCCACCTTCCAGCAGAT-3´ R5´- TCACCTTCACCGTTCCAGT-3´ |
| GAPDH | NM_001034034 | F5´- AATGGAGCCATCACCATC-3´ R5´-GTGGTTCACGCCCATCACA-3´ |
| Additional File Table S10. Sequence specific primers used for qRT-PCR analysis. | | |
